# Local Resting Ca^2+^ Controls the Scale of Astroglial Ca^2+^ Signals

**DOI:** 10.1016/j.celrep.2020.02.043

**Published:** 2020-03-10

**Authors:** Claire M. King, Kirsten Bohmbach, Daniel Minge, Andrea Delekate, Kaiyu Zheng, James Reynolds, Cordula Rakers, Andre Zeug, Gabor C. Petzold, Dmitri A. Rusakov, Christian Henneberger

**Affiliations:** 1 Institute of Neurology, University College London, London, UK; 2 Institute of Cellular Neurosciences, Medical Faculty, University of Bonn, Bonn, Germany; 3 German Center for Neurodegenerative Diseases (DZNE), Bonn, Germany; 4 Cellular Neurophysiology, Hannover Medical School, Hannover, Germany; 5 Department of Neurology, University Hospital Bonn, Bonn, Germany

**Keywords:** brain, astrocytes, calcium signalling, mechanism, calcium stores, fluorescence microscopy, quantitative, hippocampus, cortex, in vivo, locomotion

## Abstract

Astroglia regulate neurovascular coupling while engaging in signal exchange with neurons. The underlying cellular machinery is thought to rely on astrocytic Ca^2+^ signals, but what controls their amplitude and waveform is poorly understood. Here, we employ time-resolved two-photon excitation fluorescence imaging in acute hippocampal slices and in cortex *in vivo* to find that resting [Ca^2+^] predicts the scale (amplitude) and the maximum (peak) of astroglial Ca^2+^ elevations. We bidirectionally manipulate resting [Ca^2+^] by uncaging intracellular Ca^2+^ or Ca^2+^ buffers and use ratiometric imaging of a genetically encoded Ca^2+^ indicator to establish that alterations in resting [Ca^2+^] change co-directionally the peak level and anti-directionally the amplitude of local Ca^2+^ transients. This relationship holds for spontaneous and for induced (for instance by locomotion) Ca^2+^ signals. Our findings uncover a basic generic rule of Ca^2+^ signal formation in astrocytes, thus also associating the resting Ca^2+^ level with the physiological “excitability” state of astroglia.

## Highlights

• Ca^2+^ signals in brain astroglia are controlled by the local basal [Ca^2+^]
• A high basal [Ca^2+^] is associated with higher peak level but lower amplitude
• This rule applies to Ca^2+^ signals *in vitro* and *in vivo*
• Changing basal Ca^2+^ affects peak and amplitude of Ca^2+^ signals accordingly

## Graphical Abstract

**Figure:**
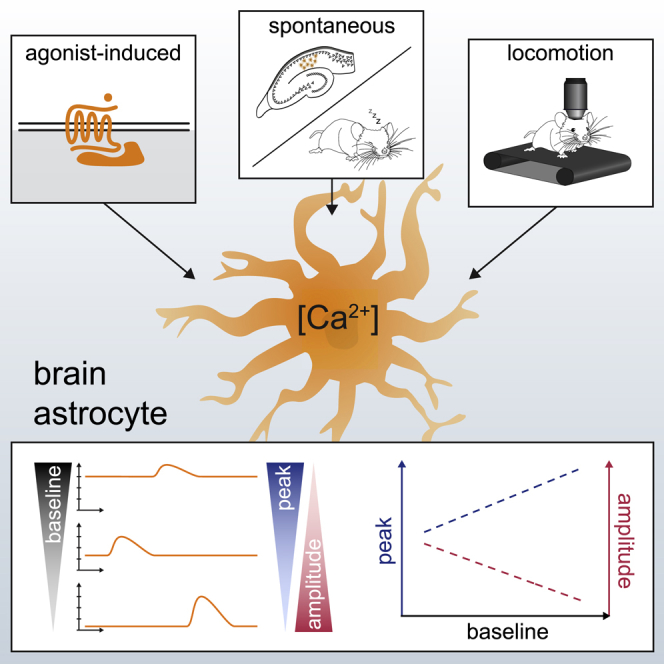

## Teaser

King et al. show that, in brain astroglia, a high resting [Ca^2+^] increases the maximum level but decreases the amplitude of local Ca^2+^ signals. This fundamental relationship holds over a wide range of preparations, ranging from hippocampal astrocytes in acute slices to locomotion-associated Ca^2+^ signals in the awake animal.

## Introduction

Astroglial cells are an abundant, electrically non-excitable cell type that contribute to brain function via various mechanisms, including the maintenance of extracellular ion homeostasis, neurotransmitter uptake, neurovascular coupling, and reciprocal neuron-astroglia signaling. The key intracellular messenger in astroglia is Ca^2+^. A multitude of signaling pathways enable astroglia to convert neuronal activity into cytosolic Ca^2+^ increases, which often involve Ca^2+^ stores (Bazargani and Attwell, 2016, Shigetomi et al., 2016, Volterra et al., 2014). In turn, an increase in intracellular Ca^2+^ triggers diverse cellular responses, such as Ca^2+^-dependent neurotransmitter release from astroglia and neurovascular coupling (Araque et al., 2014, Nuriya and Hirase, 2016, Perea et al., 2009, Petzold and Murthy, 2011, Rusakov et al., 2014).

Recent advances in Ca^2+^ imaging have helped to reveal that astroglial Ca^2+^ transients vary extensively in their magnitude, intracellular location, and spatiotemporal dynamics (Bindocci et al., 2017, Di Castro et al., 2011, Kanemaru et al., 2014, Srinivasan et al., 2015), suggesting that astrocytes are fully equipped to provide graded, wide-bandwidth control of their Ca^2+^-dependent actions. Indeed, it has long been shown that the rate of exocytosis and the fraction of released vesicles increases with greater Ca^2+^ mobilization in cultured astroglia (Kreft et al., 2004, Pryazhnikov and Khiroug, 2008) and that astroglial glutamate release depends on the magnitude of Ca^2+^ entry (Parpura and Haydon, 2000). Graded effects of astrocytic Ca^2+^ elevations on neural function have since been reported in various brain regions, *in situ* and *in vivo* (reviewed in Bazargani and Attwell, 2016, Volterra et al., 2014). Similarly, graded astroglial [Ca^2+^] signals can control blood vessel diameters and blood flow (Lind et al., 2013, Mishra et al., 2016, Mulligan and MacVicar, 2004).

Clearly, the amplitude and extent of transient [Ca^2+^] changes are driven by multiple molecular mechanisms operating in astrocytes, such as Ca^2+^ release from intracellular stores, mitochondria, or via channel-mediated Ca^2+^ entry (Agarwal et al., 2017, Araque et al., 2014, Bazargani and Attwell, 2016, Verkhratsky and Nedergaard, 2018, Volterra et al., 2014). Given the multiplicity of astrocytic cellular mechanisms contributing to Ca^2+^ signaling, the question arises whether there are any unifying physiological principles that shape the waveform of Ca^2+^ elevations in astroglia.

Intriguingly, several lines of experimental evidence suggest that the cytosolic resting [Ca^2+^] could be a key player here. First, resting [Ca^2+^] in astroglia can be modulated by neuronal activity in rat somatosensory cortex slices (Mehina et al., 2017) and hippocampal slice cultures (Jackson and Robinson, 2015) and by dopamine in acute hippocampal slices (Jennings et al., 2017). In cortical astroglia *in vivo*, basal [Ca^2+^] shows a heterogeneous distribution pattern within and among cells (Zheng et al., 2015) and is globally elevated in a mouse model of Alzheimer’s disease (Kuchibhotla et al., 2009).

Second, Ca^2+^ entry into the astroglial cytosol from the endoplasmic reticulum (ER) via inositol 1,4,5-trisphosphate receptors (IP3Rs) is determined by the receptor opening probability, which has a bell-shaped dependence on cytosolic [Ca^2+^] (Bezprozvanny et al., 1991, Foskett et al., 2007). Thus, an increase of the resting [Ca^2+^] from low values could boost IP3R-dependent astroglial [Ca^2+^] transients, whereas relatively high resting [Ca^2+^] may dampen their amplitude.

Third, the resting [Ca^2+^] sets the degree to which endogenous intracellular Ca^2+^ buffers are occupied by Ca^2+^. For instance, lowering the resting [Ca^2+^] sharply increases the proportion of endogenous buffers that can effectively curtail the extent of free cytosolic [Ca^2+^] transients and vice versa. Indeed, in neurons, Ca^2+^ buffer saturation has been shown to contribute to the facilitation of Ca^2+^-dependent release during repetitive stimulation (Klingauf and Neher, 1997, Scott and Rusakov, 2006, Vyleta and Jonas, 2014). This suggests that an increase of astroglial resting [Ca^2+^] could partly saturate endogenous Ca^2+^ buffers and thereby increase the amplitude of cytosolic Ca^2+^ transients.

Thus, our hypothesis was that resting Ca^2+^ levels could be an important determinant of Ca^2+^ signal generation in astrocytes. To test this hypothesis, we investigated the relationship between astroglial resting [Ca^2+^] and [Ca^2+^] transients *in vitro* and *in vivo* by employing ratiometric fluorescence intensity measurements of genetically encoded Ca^2+^ indicators and fluorescence lifetime imaging (FLIM) of organic Ca^2+^ indicators. We combined these measurements with photolytic release of Ca^2+^ and Ca^2+^ buffers inside individual astrocytes to establish a causal relationship between astroglial resting [Ca^2+^] and astroglial [Ca^2+^] transients.

## Results

### Resting [Ca^2+^] Predicts Maximum (Peak) and Amplitude of Evoked Astroglial Ca^2+^ Transients *In Situ*

The Ca^2+^ dependence of the fluorescence lifetime of dyes from the Oregon Green BAPTA family was used to accurately measure [Ca^2+^] in astroglia *in situ* and *in vivo* by performing FLIM (Agronskaia et al., 2004, Kuchibhotla et al., 2009, Wilms et al., 2006, Zheng et al., 2015). We chose Oregon Green 488 BAPTA-2 (OGB2) because of its relatively high molecular weight of ∼1.5 kDa, which makes OBG2 less likely to escape via gap junctions into neighboring astrocytes, thereby keeping its concentration high in the small peripheral branches of astrocytes.

**Figure 1:**
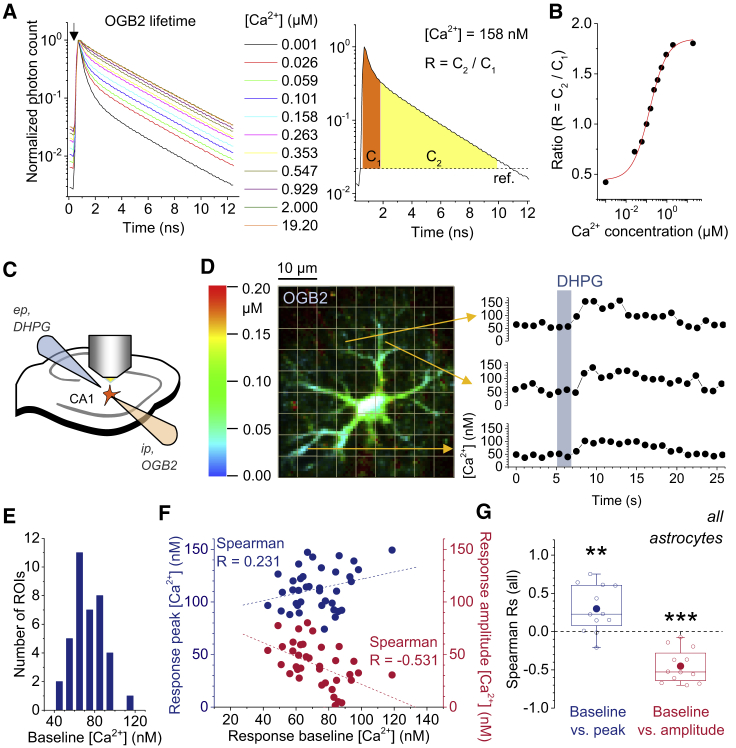
Variable Subcellular and Intercellular Astroglial Resting/Baseline [Ca^2+^] Determines Ca^2+^ Transient Properties (A) The fluorescence lifetime of the Ca^2+^ indicator OGB2 depends on [Ca^2+^] (left panel; cuvette). Shown is the normalized photon count rate measured by time-correlated single-photon counting (TCSPC) relative to the laser pulse (arrow). The [Ca^2+^] dependence of OGB2 lifetime was quantified (right panel; example for [Ca^2+^] = 158 nM) by calculating the ratio (R) of the number of photons (C) detected in two time windows (C1, orange; C2, yellow). (B) The relationship of the ratio R and [Ca^2+^] was approximated by a logistic function to translate ratios obtained in Ca^2+^ imaging experiment into Ca^2+^ concentrations. (C) Schematic of the recording configuration for acute slices. Recordings were performed in the *stratum radiatum* of the hippocampal CA1 region. Individual astroglial cells were filled with the Ca^2+^-sensitive fluorescent dye OGB2 via the whole-cell patch-clamp pipette (ip, intracellular pipette). Astroglial Ca^2+^ responses were evoked by pressure application of extracellular solution containing the mGluR agonist DHPG (300 μM) and Alexa Fluor 647 (3 μM; for visualizing the puff) in the immediate vicinity of the cell using an extracellular pipette (ep). (D) Illustration of a sample recording. Image of an OGB2-filled astrocytes (left panel; [Ca^2+^] color-coded according to scale; brightness corresponds to fluorescence intensity; grid outlines regions of interest [ROIs]). Application of DHPG (right panel) by a short pressure pulse reliably induced [Ca^2+^] transients throughout the astrocyte (3 sample ROIs as indicated by arrows). (E) The resting [Ca^2+^] varies considerably across the ROIs (example from D). (F) The relationship between the [Ca^2+^] before the DHPG puff and the peak [Ca^2+^] of the transients (blue) and the transient amplitude (red) was quantified by calculating the Spearman rank correlation coefficient (R) (38 ROIs; same cell as in D and E). (G) Over all experiments, the Rs were positive for the baseline [Ca^2+^] and transient peak [Ca^2+^] (p = 0.0060) and negative for baseline [Ca^2+^] and the transient amplitude (p < 0.0001; n = 11 for both; one-population two-sided Student’s t tests). Each point represents a recording from one astrocyte.

First, OGB2 lifetime measurements (time-correlated single photon counting [TCSPC]) were calibrated for free Ca^2+^ (Figure 1A). For analyses, the time-resolved OGB2 fluorescence decay was split in two time windows (C_1_ and C_2_), in which the numbers of detected photons were counted before their ratio was taken (photon count ratio; Figure 1A, right panel). This photon count ratio displayed a strong dependence on [Ca^2+^]: the advantages of this ratiometric measure over the conventional multi-exponential approximation of lifetime decay have been demonstrated (Zheng et al., 2015). It was therefore used to quantify OGB2 fluorescence decay and to translate it into [Ca^2+^] (Figures 1B and S1; STAR Methods).

To monitor astroglial Ca^2+^ signaling in CA1 *stratum radiatum* of the hippocampus, we loaded OGB2 (200 μM) via a whole-cell patch pipette into astrocytes. After an equilibration period of 10–20 min, astroglial Ca^2+^ transients were evoked by pressure application of the mGluR agonist DHPG from a pipette placed immediately adjacent to the astrocyte territory while collecting OGB2 fluorescence (Figure 1C). We used mGluR activation because it reliably induces astroglial Ca^2+^ signaling in the hippocampus (Porter and McCarthy, 1996, Sherwood et al., 2017, Tang et al., 2015). For analyses, a square grid of the regions of interest (ROIs) was automatically defined (Figure 1D, left); the time course of [Ca^2+^] was determined in each ROI (Figure 1D; STAR Methods); and the resting [Ca^2+^] before the DHPG application, the peak [Ca^2+^] of the response, and their difference, i.e., the response amplitude, were calculated. Notwithstanding the well-known complexities of Ca^2+^ signal propagation in astrocytes, the ROI-based approach was specifically selected to report the relationship between local resting Ca^2+^ and the amplitude and peak of the local transient, regardless of its origin.

The baseline/resting [Ca^2+^] displayed considerable variability between regions of an individual cell (Figure 1E), with an average coefficient of variation (CV) of 0.36 ± 0.033 (n = 12) and an average mean of 62.7 ± 9.64 nM (standard deviation 33.4 nM; n = 12), consistent with previous observations (Zheng et al., 2015). If these variations of baseline [Ca^2+^] have a role in determining the amplitude of [Ca^2+^] transients or their peak [Ca^2+^], DHPG-induced responses should depend on the local baseline [Ca^2+^]. To identify an association, if any, between measured [Ca^2+^] parameters without any assumptions about their exact mathematical relationships, we used the standard Spearman’s rank correlation (see also Quantification and Statistical Analysis in STAR Methods). We calculated the Spearman’s rank correlation coefficient (R) between the resting [Ca^2+^], the peak [Ca^2+^] (maximum elevation), and the amplitude (concentration increase with respect to the baseline/resting level) to capture dependencies across a recorded astrocyte (Figure 1F). On average, across all cells, the resting/baseline [Ca^2+^] and the peak [Ca^2+^] were positively correlated, whereas a negative correlation between baseline [Ca^2+^] and the amplitude of DHPG-induced Ca^2+^ transients was detected (Figures 1G and S3A–S3C for a direct comparison of peaks and amplitudes between high and low baseline [Ca^2+^]). Where detected, progressive decreases in Ca^2+^ transient amplitudes were not due to saturation of OGB2, because the maximum peak [Ca^2+^] observed in a cell, averaged across the entire population, was 191.2 ± 38.2 nM. This is equivalent to photon count ratios about halfway between their minimum and maximum (Figure 1B; ∼57%) and well below the estimated affinity of OGB2 for Ca^2+^ (580 nM; ThermoFisher). We also tested whether variations of the resting [Ca^2+^] could influence the kinetics of Ca^2+^ transient, which was however not the case (Figure S2). Thus, inside individual astroglia, a higher resting [Ca^2+^] predicts a higher peak [Ca^2+^] and lower amplitudes of Ca^2+^ transients triggered by mGluR activation.

### Resting [Ca^2+^] versus Intracellular Location

We next asked whether this finding can be explained by local astrocyte morphology. Because the fine structural details cannot be resolved by diffraction-limited two-photon excitation microscopy (Heller and Rusakov, 2015), we used the local fraction of tissue volume (volume fraction [VF]) occupied by astrocyte branches as a measure of local morphology (Medvedev et al., 2014, Savtchenko et al., 2018). The local VF was calculated for each ROI by normalizing the local Alexa Fluor 594 fluorescence intensity to the somatic values. This VF is highest at the soma (100%), low if the ROI is occupied by thin astrocyte branches, and intermediate if ROIs contain larger branches and thin processes. For each astrocyte, we then compared the local VF with the baseline [Ca^2+^], the transient peak [Ca^2+^], and the amplitude. The average R between VF and baseline [Ca^2+^] indicated no significant correlation (mean R = 0.106 ± 0.0864; n = 11; p = 0.247; one-population Student’s t test). This result suggested that the baseline [Ca^2+^] is not primarily determined by local astrocyte morphology. In contrast, we found negative correlations between the local VF and peak [Ca^2+^] (R = −0.234 ± 0.0842; p = 0.0191; n = 11; one-population Student’s t test) and between VF and the amplitude (R = −0.285 ± 0.0779; p = 0.00435; n = 11; one-population Student’s t test). The latter observation indicates that, independently of baseline [Ca^2+^], the amplitude and peak [Ca^2+^] of mGluR-dependent transients are higher when the local VF is low, i.e., in the small astrocyte branches.

These experiments establish that astroglial compartments with higher resting [Ca^2+^] display transients with a higher peak [Ca^2+^], but lower amplitudes, within individual cells. Higher baseline [Ca^2+^] could decrease Ca^2+^ entry into the astrocyte cytosol by reducing Ca^2+^ influx either from extracellular space or from Ca^2+^ stores. Theory dictates that 50 nM changes in basal [Ca^2+^] lead to only <10% changes in its extracellular driving force (Figure S3D). In contrast, a significant decrease of the driving force for Ca^2+^ entry from internal Ca^2+^ stores appears plausible, depending on the estimates of the Ca^2+^ store membrane potential and intra-store [Ca^2+^] (Figures S3E and S3F). In addition, full equilibration of Ca^2+^ between the astrocyte cytosol and intracellular Ca^2+^ stores during a cytosolic Ca^2+^ transient could lead to a decrease of Ca^2+^ transient amplitudes at high-baseline basal [Ca^2+^] (Figure S4). This would again depend on the exact relative volumes and relative [Ca^2+^] between cytosol and intracellular Ca^2+^ stores.

Irrespective of the underlying mechanism, these results demonstrate a clear correlation between resting Ca^2+^ and evoked Ca^2+^ transients. However, they do not establish the underlying causality. Therefore, we next asked whether controlled manipulation of resting [Ca^2+^] would predictably affect transient Ca^2+^ signaling.

### Induced Changes in Resting [Ca^2+^] Bidirectionally Regulate Evoked Ca^2+^ Signals Transients *In Situ*

**Figure 2:**
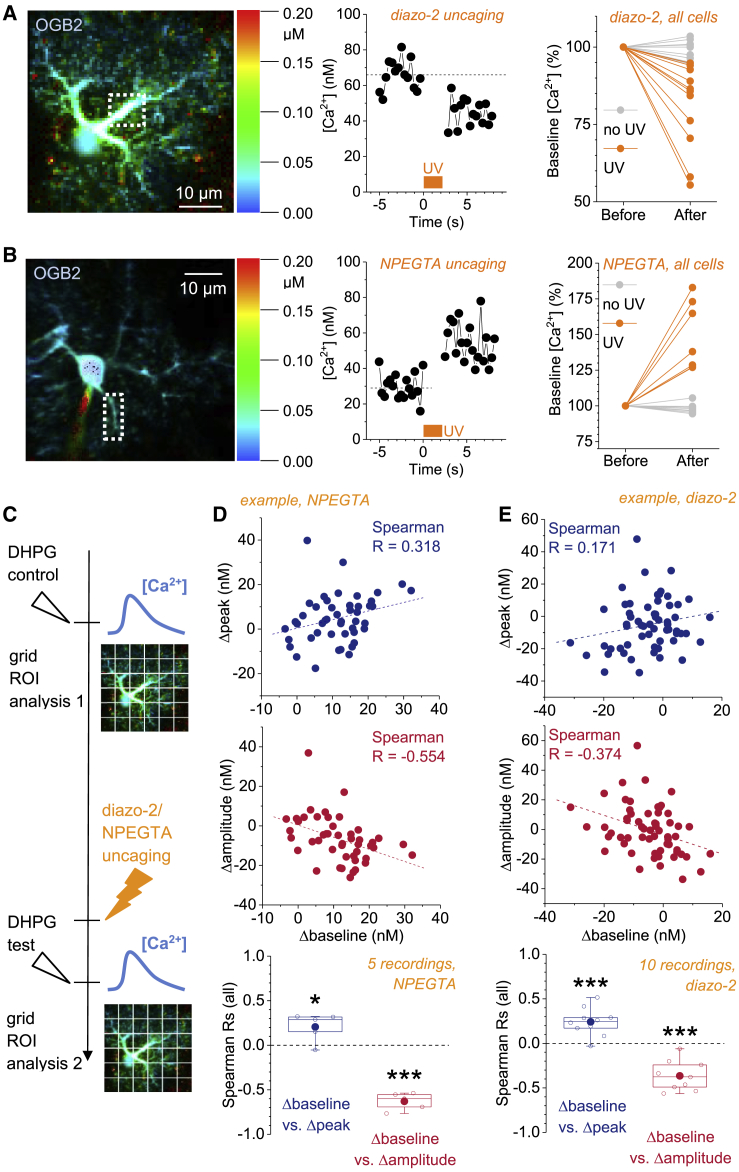
Induction of Bidirectional Changes of the Local Astroglial Baseline [Ca^2+^] Modifies Evoked Ca^2+^ Transients (A) The basal [Ca^2+^] concentration was reliably lowered by photolysis of diazo-2 (included in the intracellular patch clamp solution). Example of an astroglial Ca^2+^ landscape before UV illumination is shown (left panel; dashed box represents example ROI; FLIM Ca^2+^ imaging throughout). The [Ca^2+^] decreased by ∼20 nM upon UV illumination (same ROI, middle panel; orange bar indicates UV exposure). UV exposure significantly reduced the overall Ca^2+^ concentration in the astrocyte by 19.3% ± 4.20% (n = 11; p < 0.001; one-population Student’s t test), whereas no change was detected by including diazo-2 only (without UV; 1.26% ± 0.953%; n = 11; p = 0.218; one-population Student’s t test). In experiments with UV photolysis of diazo-2, the time window of analysis corresponds to the baseline period of the 2^nd^ DHPG puff (see C and E). (B) Photolysis of the Ca^2+^ cage NP-EGTA increased the baseline [Ca^2+^]. Same layout as in (A). UV illumination significantly increased the overall baseline [Ca^2+^] in the astrocyte (UV: +46.1% ± 8.3%, n = 8, p < 0.001, one-population Student’s t test; no UV: −1.86% ± 1.21%, n = 8, p = 0.169, one-population Student’s t test). In experiments with UV photolysis of NP-EGTA, the time window of analysis corresponds to the baseline period of the 2^nd^ DHPG puff (see C and D). (C) Timeline of the experiment. A first Ca^2+^ transient was evoked by pressure application of DHPG. [Ca^2+^] baseline, peak, and amplitude were determined for every ROI in a grid, like in Figure 1 (example image here, not experimental data). Then, either diazo-2 or NP-EGTA was photolysed by UV illumination. A second Ca^2+^ transient was evoked 6–10 s after photolysis. Again, [Ca^2+^] baseline, peak, and amplitude were determined for every ROI in a grid. For each ROI with a DHPG response bigger than 6 nM, we then calculated the change of [Ca^2+^] baseline, peak, and amplitude between before and after uncaging (after–before, Δbaseline, Δpeak, and Δamplitude). In control experiments without UV photolysis, the amplitude of the DHPG-induced Ca^2+^ transients changed by 1.62% ± 12.1% from one DHPG puff to the next (n = 7; p = 0.75; paired Student’s t test). (D) NPEGTA uncaging. The changes of the pre-event baseline [Ca^2+^], the peak [Ca^2+^], and the amplitude were calculated (as in D, Δbaseline, Δpeak, and Δamplitude) and correlated in each cell (R). Top and middle panels: an example recording displays changes of peak and amplitude versus baseline [Ca^2+^] changes within individual ROIs from a single recording. An overall increase of baseline [Ca^2+^] from 27.1 nM to 38.5 nM was induced in this cell. Bottom panel: Rs show that increases of baseline [Ca^2+^] are correlated with an increased peak [Ca^2+^] and a decreased amplitude of DHPG-evoked Ca^2+^ transients (n = 5; Δbaseline versus Δpeak R = +0.205 ± 0.0718, p = 0.0458; Δbaseline versus Δamplitude R = −0.630 ± 0.0435, p < 0.001; one-population Student’s t tests). Each data point represents a recording from one astrocyte. (E) Diazo-2 uncaging. The changes of the pre-event baseline [Ca^2+^], the peak [Ca^2+^], and the amplitude were calculated (as in D, Δbaseline, Δpeak, and Δamplitude) and correlated in each cell (R). Top and middle panels: an example recording displays changes of peak and amplitude versus baseline [Ca^2+^] changes within individual ROIs from a single recording. A reduction of overall baseline [Ca^2+^] from 35.8 nM to 31.0 nM was induced in this example. Bottom panel: Rs for all experiments show that a decrease of baseline [Ca^2+^] is accompanied by a decreased peak [Ca^2+^] and increased amplitude of DHPG-evoked Ca^2+^ transients (n = 10; Δbaseline versus Δpeak R = +0.241 ± 0.0491, p < 0.001; Δbaseline versus Δamplitude R = −0.365 ± 0.0505, p < 0.001; one-population Student’s t tests). Each data point represents a recording from one astrocyte.

Two experimental approaches were used to manipulate the baseline [Ca^2+^] of astrocytes within the observed physiological range (Figures 1E and 1F). To lower the baseline [Ca^2+^], the light-sensitive Ca^2+^ chelator diazo-2 (2.5 mM) was added to the intracellular solution: UV photolysis of diazo-2 drastically increases its affinity for Ca^2+^ (decrease of K_D_ from ∼2.2 μM to ∼73 nM; Adams et al., 1989). A brief UV exposure of diazo-2-filled astrocytes indeed robustly decreased the baseline [Ca^2+^] (Figure 2A). In a complementary set of tests, we sought to increase the baseline [Ca^2+^] by adding an equilibrated mixture of the photolysable Ca^2+^ chelator NP-EGTA (5 mM) and CaCl_2_ (3 mM) to the intracellular solution and using UV illumination to release Ca^2+^ (Fellin et al., 2004). This reliably induced a significant increase of the baseline [Ca^2+^] in astrocytes (Figure 2B).

We then asked how the evoked Ca^2+^ transients were affected by manipulating the baseline [Ca^2+^] (Figure 2C). From our previous recordings, we expected that increasing the local baseline [Ca^2+^] should increase the [Ca^2+^] transient peak and decrease the amplitude. To test this, Ca^2+^ transients evoked by DHPG before and after changing the baseline [Ca^2+^] were compared, in each recording and each ROI (Figures 1D and 2C). The corresponding changes of the baseline [Ca^2+^], the transient peak [Ca^2+^], and the amplitude were documented. In each astrocyte, the R between these changes was computed (see Figure 2D, top and middle panels, one-cell example). In recordings using NP-EGTA, we did find a positive correlation between the changes of baseline [Ca^2+^] and transient peak [Ca^2+^], and a negative correlation between the changes of baseline [Ca^2+^] and the amplitude (Figure 2D, bottom panel). Similar results were obtained in recordings employing photolysis of diazo-2. Here, a decrease of baseline [Ca^2+^] was associated with a decrease of peak [Ca^2+^] and an increase of the amplitude (Figure 2E). Note that photolysis of either diazo-2 or NP-EGTA changes the overall concentration and properties of mobile Ca^2+^ buffers throughout the cell, which may globally decrease or increase, respectively, the DHGP-induced [Ca^2+^] amplitudes and peaks. This does not affect correlations between local regions of interest within single cells as performed here.

Fully consistent with our initial observations, these findings indicate that changes in the local resting [Ca^2+^] are sufficient to alter both the peak [Ca^2+^] and the amplitude of DHPG-evoked Ca^2+^ transients: a local increase of baseline [Ca^2+^] leads to an increased peak [Ca^2+^] and decreased amplitude.

### Spontaneous Astroglial Ca^2+^ Transients Depend on Resting [Ca^2+^] *In Situ*

**Figure 3:**
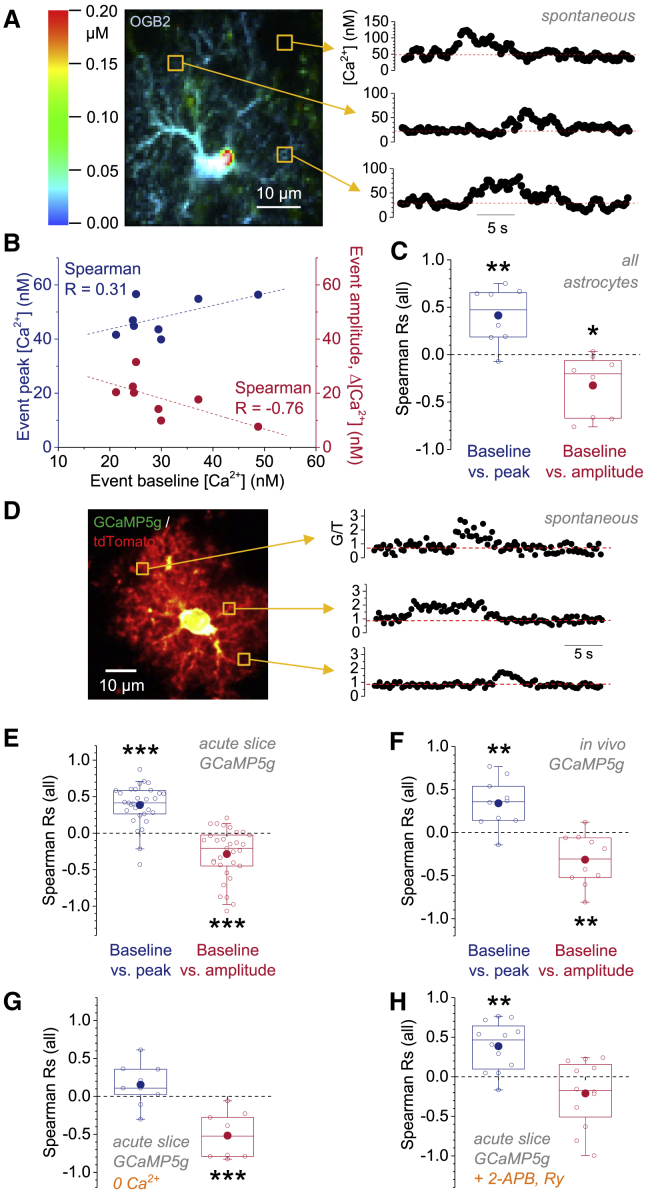
Amplitude and Peak [Ca^2+^] of Spontaneous Astroglia Ca^2+^ Transients Vary with Resting [Ca^2+^] (A) Sample FLIM Ca^2+^ recording with [Ca^2+^] landscape and three sample ROIs (left panel). Three examples of [Ca^2+^] transients from the indicated ROIs (right panel) are shown. Note that these transients did not occur simultaneously but have been roughly aligned for illustration purposes. Dashed red line indicates resting/baseline [Ca^2+^] before the event. (B) Correlation of spontaneous transient (event) baseline [Ca^2+^] and peak [Ca^2+^] (blue) and amplitude (red) for the example shown in (A). Rs are shown. Each data point represents data from an individual event and ROI. (C) Overall, Rs reveal that higher pre-event baselines [Ca^2+^] are associated with higher peak [Ca^2+^] and lower amplitudes of spontaneous transients (n = 8; baseline versus peak: R = +0.413 ± 0.106, p = 0.0059; baseline versus amplitude: R = −0.325 ± 0.114, p = 0.025; one-population Student’s t tests). Each point represents a FLIM recording from one astrocyte. (D) To confirm these findings in astrocytes not perturbed by whole-cell patch clamp, transgenic mouse lines were used that express the genetically encoded Ca^2+^ indicator GCaMP5g (G) and the fluorescent protein tdTomato (T) in astrocytes (left panel; example of tdTomato-expressing astrocyte with ROIs, boxes). Spontaneous Ca^2+^ transients were observed and quantified after calculation of the background-corrected fluorescence intensity ratio G/T. Sample Ca^2+^ transients are shown in the right panel (not occurring simultaneously; roughly aligned in time for illustration). Red dashed lines indicate pre-event baseline G_B_/T. (E) Acute slice recordings. Left: all Rs between pre-event resting G_B_/T and transient peak G_P_/T (blue; R = +0.386 ± 0.0488; ^∗∗∗^p = 8.2 × 10^−9^; one-population Student’s t test). Right: all Rs between pre-event G_B_/T and transient amplitude G_P_/T − G_B_/T after correction are shown (red; R = −0.285 ± 0.0622; ^∗∗∗^p = 7.4 × 10^−5^; one-population Student’s t test). For correction, see Figure S5. Data points represent Rs from individual cells (for both n = 31 from 31 slices from more than seven animals). (F) *In vivo* recordings of spontaneous Ca^2+^ transients from astrocytes expressing GCaMP5g and tdTomato (n = 10 from five anesthetized mice). Left: Rs calculated between pre-event baseline G_B_/T and transient peak G_P_/T are shown (blue; R = +0.340 ± 0.0875; ^∗∗^p = 0.0037; one-population Student’s t test). Right: Rs of pre-event G_B_/T and transient amplitude G_P_/T − G_B_/T after correction are shown (red; R = −0.315 ± 0.0940; ^∗∗^p = 0.0085; one-population Student’s t test). For correction, see Figure S5. Data points represent Rs from individual cells (n = 10 astrocytes from three animals). (G) Acute slice recordings of spontaneous Ca^2+^ transients in the absence of extracellular Ca^2+^ (GCaMP5g). Left: Rs calculated between pre-event baseline G_B_/T and transient peak G_P_/T are shown (blue; R = +0.153 ± 0.0908; p = 0.13; one-population Student’s t test). Right: Rs for pre-event G_B_/T and transient amplitude G_P_/T − G_B_/T after correction are shown (red; R = −0.516 ± 0.0971; ^∗∗∗^p = 7.1 × 10^−4^; one-population Student’s t test). For correction, see Figure S5. Data points represent Rs from individual cells (n = 9 recordings from slices obtained from three animals). (H) Recordings of spontaneous Ca^2+^ transients (GCaMP5g) in the presence of the IP3 receptor inhibitor 2-APB (100 μM) and ryanodine (10 μM) from acute slices. Left: Rs calculated between pre-event baseline G_B_/T and transient peak G_P_/T are shown (blue; R = +0.386 ± 0.0897; ^∗∗^p = 0.0012; one-population Student’s t test). Right: Rs of pre-event G_B_/T and transient amplitude G_P_/T − G_B_/T after correction are shown (red; R = −0.211 ± 0.121; p = 0.11; one-population Student’s t test). For correction, see Figure S5. Data points represent Rs from individual cells (n = 12 recordings from slices obtained from three animals).

Although DHPG pressure application reliably evoking timed astroglial Ca^2+^ transients, it might engage Ca^2+^ signaling cascades that differ from those endogenously active. We therefore next analyzed Ca^2+^ transients spontaneously occurring during FLIM imaging (Figure 3), without any pharmacological stimulation. Spontaneous transients were analyzed in manually selected ROIs (∼3 × 3 μm^2^) and occurred at an average frequency of 1.40 ± 0.159 events per minute (n = 7; Figure 3A). On average, the measured pre-event baseline [Ca^2+^] was 29.3 ± 11.6 nM and the peak [Ca^2+^] was 50.9 ± 15.3 nM (n = 8). Note that these are free Ca^2+^ concentration estimates in the presence of 200 μM added exogenous buffer (OGB2) and that the free cytosolic Ca^2+^ concentration may rise much further in its absence.

How do these measurements compare with those reported elsewhere? Ca^2+^ transients are often quantified by normalizing the fluorescence intensity of the Ca^2+^ indicator at the peak to its pre-event baseline value (F_P_/F_0_). Assuming that the fluorescence of the Ca^2+^-free indicator is negligible (e.g., Fluo-4), F_P_/F_0_ can be derived from the law of mass action to be [Ca^2+^]_P_ × (K_D_ + [Ca^2+^]_0_)/([K_D_ + [Ca^2+^]_P_] × [Ca^2+^]_0_), where K_D_ is the indicator’s dissociation constant and [Ca^2+^]_0_ (baseline) and [Ca^2+^]_P_ (peak) correspond to F_P_ and F_0_. Using a K_D_ of 350 nM for Fluo-4, our measurements correspond to F_P_/F_0_ values of 1.86 ± 0.118 (n = 8; range = 1.29–2.23), which is well within the range of published data, both *in vitro* and *in vivo* (Hirase et al., 2004, Navarrete and Araque, 2008).

Spontaneous Ca^2+^ transients very rarely occurred in the same region more than once during 5–10 min. We could therefore not test how the UV uncaging of Ca^2+^ and Ca^2+^ buffers affect spontaneous Ca^2+^ transients in the same ROI. Instead, we analyzed the correlations between baseline [Ca^2+^], transient peak [Ca^2+^], and the amplitude across all transients recorded in individual cells (Figures 3A and 3B). Like DHPG-evoked Ca^2+^ transients, we found that a higher resting [Ca^2+^] was associated with a higher peak [Ca^2+^] and lower amplitude (Figure 3C).

In summary, peak [Ca^2+^] and amplitude of spontaneous, endogenously generated Ca^2+^ transients display the same dependence on the pre-event local resting [Ca^2+^] as DHPG-evoked responses. This confirms that the pre-transient baseline or resting [Ca^2+^] controls astroglial [Ca^2+^] transients.

### Control of Ca^2+^ Transients by Resting [Ca^2+^] Is a Ubiquitous Phenomenon

Do our findings faithfully reflect the role of resting [Ca^2+^] in controlling Ca^2+^ signals in astrocytes unperturbed by whole-cell patch-clamp recordings and *in vivo*? Genetically encoded Ca^2+^ indicators have greatly facilitated the analysis of astroglial Ca^2+^ signaling, especially *in vivo* (Bindocci et al., 2017, Gee et al., 2014, Kanemaru et al., 2014, Shigetomi et al., 2010). Because the routinely used GCaMP-type indicators display little to no lifetime changes upon Ca^2+^ binding (Akerboom et al., 2012), they are not suitable for FLIM Ca^2+^ imaging. On their own, these Ca^2+^ indicators also do not lend themselves to quantitative comparisons of baseline Ca^2+^ and Ca^2+^ transients, because their fluorescence intensity depends on both the Ca^2+^ and the indicator concentration in a ROI. The latter varies among ROIs within the same astrocyte, because the imaged volume is only partially occupied by astrocyte cytosol and this fraction changes from one ROI to another, i.e., the local astrocyte VF varies over the cell’s territory.

Therefore, we used a mouse line that conditionally expresses the Ca^2+^ indicator GCaMP5g and the Ca^2+^-insensitive fluorescent protein tdTomato in the astroglial cytosol upon Cre recombinase expression (Gee et al., 2014). After cross-breeding with a GLASTcreERT2 mouse line (Mori et al., 2006) and tamoxifen injection, robust and astrocyte-specific expression of GCaMP5g and tdTomato was observed in the CA1 *stratum radiatum*. Because the concentration ratio of cytosol-soluble GCaMP5g and tdTomato should be constant throughout an astrocyte cytosol, we used their fluorescence intensity ratio (G/T; background corrected) to monitor intracellular [Ca^2+^] and its transient changes in an unbiased and consistent way throughout a cell.

We first performed this analysis on spontaneous Ca^2+^ transients recorded from acute slices (Figure 3D). Rs were calculated between the pre-event baseline G_B_/T and the Ca^2+^ transient peak G_P_/T and the amplitude G_P_/T − G_B_/T for each recorded cell. Because GCaMP5g fluorescence is non-linearly related to the cytosolic [Ca^2+^], we corrected Rs obtained from these recordings (Figure S5). Again, the resting [Ca^2+^] was positively correlated with the Ca^2+^ transient peak and negatively with the signal amplitude (Figure 3E). Having established these recordings, we performed *in vivo* recordings in the somatosensory cortex ∼100–200 μm below the cortical surface using a similar transgenic mouse line conditionally expressing GLASTcreER (Wang et al., 2012) and GCaMP5g/tdTomato (Gee et al., 2014) and obtained qualitatively identical results (Figure 3F). The control of astroglial Ca^2+^ transients by the local resting Ca^2+^ is therefore a robust phenomenon that can be observed using different experimental techniques, in cortex and hippocampus, *in situ* and *in vivo*.

We next investigated in acute slices whether and how Ca^2+^ entry from extracellular space and store-dependent Ca^2+^ signaling differentially affect the dependence of Ca^2+^ transients on resting Ca^2+^. In the absence of extracellular Ca^2+^, spontaneous Ca^2+^ transients are store dependent. The amplitudes of these transients displayed a strong negative correlation with the resting Ca^2+^ (Figure 3G, right panel), whereas the previously positive correlation with the peak was weaker and did not reach statistical significance (Figure 3G, left panel; p = 0.13). The opposite was found when we isolated store-independent Ca^2+^ transients by recording Ca^2+^ transients in the presence of the IP3 receptor inhibitor 2-APB and ryanodine (Figure 3H). Thus, store-dependent Ca^2+^ entry is largely responsible for the negative correlation between Ca^2+^ transient amplitudes and the resting Ca^2+^, which could arise from changes of the driving force for Ca^2+^ from Ca^2+^ stores or equilibration of Ca^2+^ between stores and cytosol (see above and Figures S3 and S4). In contrast, the amplitudes of transients mainly due to Ca^2+^ entry from extracellular space are not strongly controlled by resting Ca^2+^.

**Figure 4:**
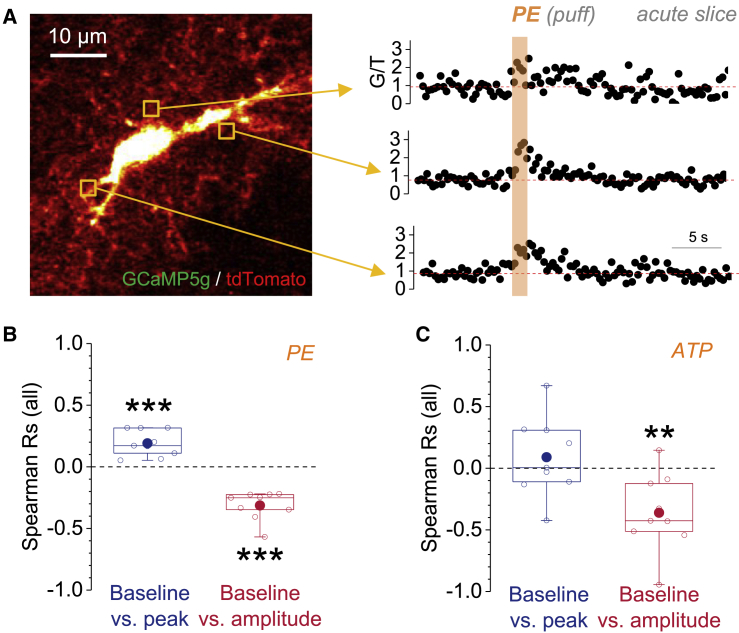
Properties of Astroglial Ca^2+^ Transients Induced by Adrenergic and Purinergic Signaling Are Controlled by the Local Resting [Ca^2+^] (A) Astroglial Ca^2+^ responses were evoked by pressure application of phenylephrine (PE) (250 μM; 80 ms) through a nearby pipette and monitored in astrocytes expressing both GCaMP5g (G) and tdTomato (T) (left panel). For analysis, the baseline, the peak, and the amplitude of drug-induced transients were quantified after calculation of the background-corrected fluorescence intensity ratio G/T (right panel; three pseudo-randomly chosen transients). (B) For PE-induced Ca^2+^ responses, the Rs were calculated between pre-event baseline G_B_/T and transient peak G_P_/T (blue; left; R = +0.190 ± 0.0354; ^∗∗∗^p = 6.6 × 10^−4^; one-population Student’s t test) and pre-event G_B_/T and transient amplitude G_P_/T − G_B_/T after correction (red; right; R = −0.314 ± 0.0388; ^∗∗∗^p = 4.0 × 10^−5^; one-population Student’s t test). For correction, see Figure S5. Data points represent Rs from individual cells (n = 10 from different slices). (C) For ATP-induced Ca^2+^ responses (5 mM; 3 × 80 ms at 4 Hz), the Rs were determined between pre-event baseline G_B_/T and transient peak G_P_/T (blue; left; R = +0.0895 ± 0.107; p = 0.43; one-population Student’s t test) and pre-event G_B_/T and transient amplitude G_P_/T − G_B_/T after correction (red; right; R = −0.361 ± 0.105; ^∗∗^p = 0.0088; one-population Student’s t test). For correction, see Figure S5. Data points represent Rs from individual cells (n = 9 from different slices).

Does this fundamental relationship hold for other types of receptor-driven and store-dependent astroglial Ca^2+^ signaling? Activation of α1 adrenoreceptors induces robust and large-scale Ca^2+^ responses in hippocampal and cortical astrocytes (Ding et al., 2013, Duffy and MacVicar, 1995). Because the α1-mediated Ca^2+^ responses in the hippocampus were previously shown to be store dependent (Duffy and MacVicar, 1995), we expected a negative correlation between the resting Ca^2+^ and their amplitude. This prediction was tested by pressure application of phenylephrine (PE) via a nearby pipette onto GCaMP5g/tdTomato-expressing astrocytes in acute slices (Figure 4A), which reliably evoked widespread Ca^2+^ responses in astrocytes. We analyzed Ca^2+^ transients in pseudo-randomly chosen ROIs and found, as predicted, that the resting Ca^2+^ was overall negatively correlated with the amplitude of Ca^2+^ transients and positively with their peak (Figure 4B). Purinergic receptor signaling is another potent trigger of Ca^2+^ signals in hippocampal astrocytes (Bowser and Khakh, 2004, Fields and Burnstock, 2006, Porter and McCarthy, 1995). We therefore tested whether ATP-induced Ca^2+^ transients are also controlled by the resting Ca^2+^. Pressure injection of ATP reliably evoked large-scale astrocyte Ca^2+^ transients, whose amplitude was negatively correlated with resting Ca^2+^, whereas no statistically significant correlation was found between the peak of Ca^2+^ transients and the resting Ca^2+^ (Figure 4C). Overall, correlations for ATP-induced transients were more variable compared to those with PE and DHPG. Potential explanations are that astrocytes can express a variety of ionotropic and metabotropic purine receptors with varying affinity for purines (Fields and Burnstock, 2006) and that applied ATP may be quickly degraded, thus leading to the uncontrolled recruitment of several pathways of Ca^2+^ entry into the astrocytic cytosol. Regardless of that variability, we found a strong negative correlation between the resting Ca^2+^ and the Ca^2+^ transient amplitude.

In summary, the local resting Ca^2+^ of astrocytes controls amplitude and peak of their Ca^2+^ transients irrespective of whether they occur spontaneously or whether they are driven by glutamatergic, adrenergic, and purinergic receptor activation. Store-dependent and independent Ca^2+^ signaling contribute differentially to this relationship.

### Both Spontaneous and Locomotion-Driven Astroglial Ca^2+^ Transients *In Vivo* Are Controlled by the Resting Ca^2+^

**Figure 5:**
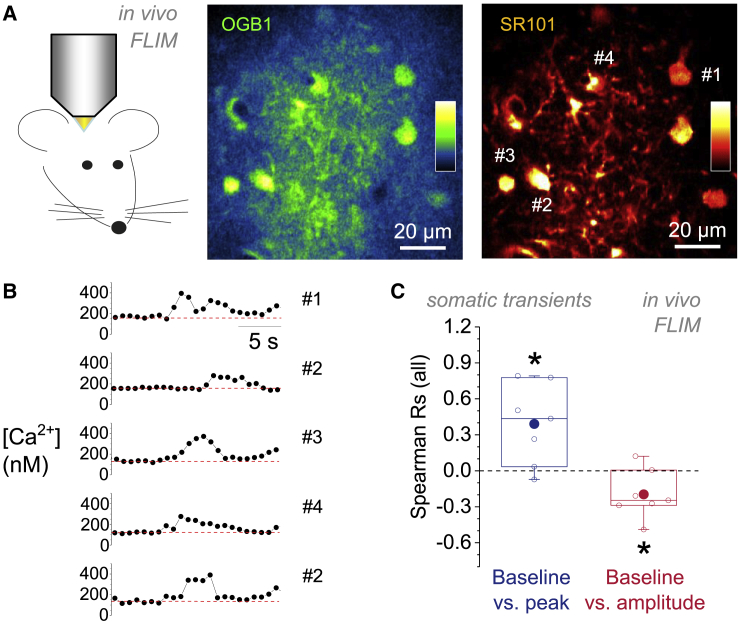
*In Vivo* Fluorescence Lifetime Imaging Confirms a Role of Resting [Ca^2+^] for Setting Properties of Astroglial Ca^2+^ Transients (A) *In vivo* FLIM Ca^2+^ recordings using OGB1 (middle panel) in SR101-labeled astrocyte somata (right panel). Examples show fluorescence intensities. Insets represent color code from minimum (bottom) to maximum (top) fluorescence intensity. Labeled cells correspond to sample traces shown in (E). (B) Sample events detected in the cells nos. 1, 2, 3, and 4 in (A) (two events from no. 2). Events did not occur simultaneously, aligned in time for illustration. Dashed red lines indicate baseline [Ca^2+^]. (C) Recordings of spontaneous somatic transients from seven separate experiments were obtained and analyzed. For each experiment, R was calculated between the pre-event resting [Ca^2+^] and the transient peak [Ca^2+^] and between the baseline [Ca^2+^] and the transient amplitude as before. Left: Rs between baseline [Ca^2+^] and peak were positive (blue; R = 0.391 ± 0.127; ^∗^p = 0.022). Right: Rs between Ca^2+^ transient baseline and amplitude are shown (red; R = −0.197 ± 0.0764; ^∗^p = 0.042). Data points represent Rs from individual recordings.

To further corroborate our findings *in vivo*, we next undertook *in vivo* FLIM Ca^2+^ imaging experiments in anesthetized rats in which astrocytes in the same brain region were bulk loaded with OGB1 and counter-stained with SR101 for identification (Zheng et al., 2015). We restricted the analysis to the somatic region of astrocytes to avoid recording from OGB1-labeled neuronal or other structures in the densely packed neuropil (Figures 5A and 5B). Within a typical recording time of 300 s, we rarely observed more than two to four somatic transients in single astrocytes. This precludes the use of the R on single-cell data. We therefore pooled [Ca^2+^] transients from all cells in a recording session before analysis so that each dataset was recorded under the same experimental conditions and with the same neuronal network activity. Astroglial resting [Ca^2+^] was again positively correlated with the peak [Ca^2+^] of spontaneous transients and negatively with their amplitudes (Figure 5C).

**Figure 6:**
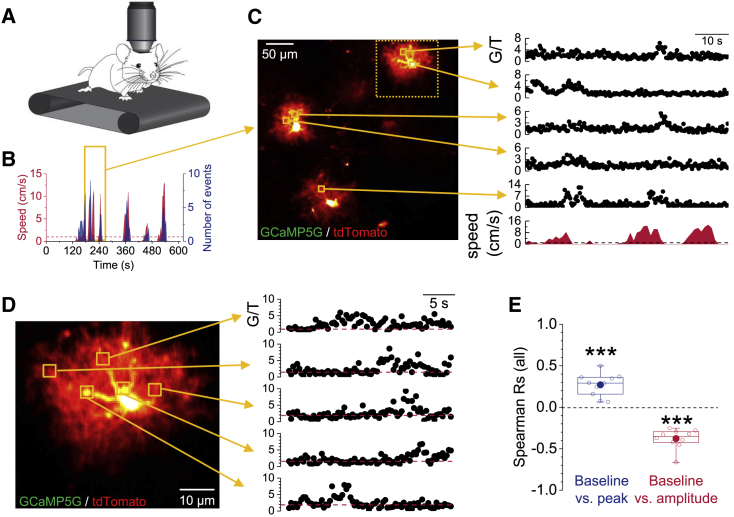
Locomotion-Associated Astroglial Ca^2+^ Transients Are Shaped by the Local Resting [Ca^2+^] (A) Schematic of recording astroglial GCaMP5g (G) and tdTomato (T) fluorescence from the somatosensory cortex in awake mice. (B) Example of the correlation between movement speed (red) and the frequency of astrocyte Ca^2+^ transients (blue; time bins of 3 s). Orange box indicates time window corresponding to examples of Ca^2+^ transients shown in (C). Dashed red line indicates speed threshold of 1 cm/s for analysis. (C) Sample view field (left panel). Ca^2+^ transients (G/T) from five sample ROIs (right panel) corresponding movement speed (bottom right panel). Note the appearance of Ca^2+^ transients time locked to movement. Ca^2+^ transients were considered to be locomotion associated if their peak occurred within a 6-s time window around a movement with a speed higher than 1 cm/s (~90% of all detected transients). Dashed orange box indicates single cell example shown in (D). (D) Single-cell example traces as in (C). Shown events did not occur simultaneously, roughly aligned in time for illustration purposes. See Figure S6 for another example. (E) For locomotion-associated Ca^2+^ responses, Rs for each recording were determined between pre-event baseline G_B_/T and transient peak G_P_/T (blue; left; R = +0.270 ± 0.0435; ^∗∗∗^p = 1.6 × 10^−4^; one-population Student’s t test) and pre-event G_B_/T and transient amplitude G_P_/T − G_B_/T after correction (red; right; R = −0.378 ± 0.0376; ^∗∗∗^p = 3.4 × 10^−6^; one-population Student’s t test). For correction, see Figure S5. Data points represent Rs from individual recordings (n = 10 recordings from four different animals).

Because the *in vivo* experiments above were done in anesthetized animals, and because anesthesia can alter astroglial Ca^2+^ signaling (Thrane et al., 2012), we performed further experiments in awake mice, in which some cortical astrocytes in the primary somatosensory cortex expressed GCaMP5g and tdTomato. It was previously discovered that astrocytes display locomotion-associated Ca^2+^ transients in awake mice (Dombeck et al., 2007). To establish whether these Ca^2+^ transients related to physiological activity are controlled by the local astroglial resting Ca^2+^, we allowed awake mice to run on a treadmill during two-photon fluorescence imaging (Figures 6A–6C). These locomotion-associated Ca^2+^ transients accounted for ∼90% of all detected Ca^2+^ transients and frequently spread throughout individual astrocytes. To correlate their amplitudes and peaks with the resting Ca^2+^, we again pseudo-randomly chose ROIs for analysis. On the subcellular level, all three parameters displayed considerable variability (CV of amplitudes 0.43 ± 0.03, of baselines 0.43 ± 0.02, and of peaks 0.35 ± 0.03; n = 10). Again, the resting Ca^2+^ level correlated negatively with the amplitude and positively with the peak of Ca^2+^ transients (Figures 6D and 6E).

Thus, our observations in live animals confirm that the local resting [Ca^2+^] is a key regulator of astroglial Ca^2+^ signals. Our results demonstrate that higher resting [Ca^2+^] levels increase the peaks while lowering the amplitudes of Ca^2+^ transients and vice versa.

## Discussion

In this study, we set out to establish how the variability of the local resting [Ca^2+^] in astroglia determines the properties of Ca^2+^ signals. Using FLIM of OGB2 loaded via the patch pipette into individual astrocytes, we observed resting [Ca^2+^] concentrations of 62.7 ± 33.4 nM (mean ± SD), which is in line with previous studies (Kuchibhotla et al., 2009, Parpura and Haydon, 2000, Zheng et al., 2015). Within individual cells, the resting [Ca^2+^] varied considerably between sub-compartments (average CV ∼0.4).

We then asked whether the magnitude of evoked Ca^2+^ transients depends on the local resting [Ca^2+^] using FLIM of OGB2-loaded astroglia. Here, we used activation of metabotropic glutamate receptors (DHPG), a robust method to induce astroglial Ca^2+^ signaling in the hippocampus at various stages of development (Porter and McCarthy, 1996, Sherwood et al., 2017, Tang et al., 2015). The peak [Ca^2+^] of evoked Ca^2+^ signals was positively correlated with the basal [Ca^2+^] before the response. In contrast, the amplitude of the response, i.e., the difference between the peak and the baseline, displayed a negative correlation with resting [Ca^2+^]. Importantly, bidirectional manipulations of the resting [Ca^2+^], by UV uncaging of either Ca^2+^ or Ca^2+^ buffers inside astroglia, affected peak and amplitude of Ca^2+^ signals in a fashion predicted by the DHPG experiments. This confirmed a causal role of the local resting [Ca^2+^] in setting peaks and amplitudes of Ca^2+^ signals. These findings were further substantiated by recordings of spontaneous Ca^2+^ transients in acute slices and *in vivo*. In addition, we found that purinergic and adrenergic Ca^2+^ transients in acute slices and locomotion-associated Ca^2+^ transients in awake mice were governed by the same principle. Thus, over a wide range of experimental protocols and preparations, the local resting [Ca^2+^] determines the properties of astroglial Ca^2+^ signals: a higher resting [Ca^2+^] leads to Ca^2+^ signals with a higher peak [Ca^2+^] but with lower amplitude and vice versa.

What could be the molecular mechanisms underlying this relationship? Our experiments demonstrate that the amplitude decrease at higher resting [Ca^2+^] is particularly prominent for store-dependent signaling. We observed for instance that, when Ca^2+^ transients were recorded in the presence of the IP3R inhibitor 2-APB and ryanodine, this inverse relationship was less pronounced and not statistically significant, whereas it was fully preserved when store-dependent signaling was isolated. Furthermore, we found this relationship for glutamatergic, purinergic, and adrenergic Ca^2+^ transients, which all have previously been attributed to store-dependent signaling (Duffy and MacVicar, 1995, Porter and McCarthy, 1995, Porter and McCarthy, 1996). IP3Rs play a major role in astroglial store-dependent Ca^2+^ signaling across brain regions *in vitro* and *in vivo*, in addition to other mechanisms, although the magnitude of their contribution varies between studies (Agarwal et al., 2017, Bazargani and Attwell, 2016, Di Castro et al., 2011, Kanemaru et al., 2014, Sherwood et al., 2017, Srinivasan et al., 2015, Tang et al., 2015, Volterra et al., 2014). One important feature of IP3Rs in the context of the present study is the dependence of their open probability on cytosolic [Ca^2+^] (Bezprozvanny et al., 1991, Foskett et al., 2007). The observed astroglial resting [Ca^2+^] was on average at 62.7 ± 33.4 nM (mean ± SD), although the peak of Ca^2+^ signals was at 118 ± 52.2 nM (mean ± SD). Within this concentration range, a rise of the resting [Ca^2+^] will increase the open probability of IP3Rs, irrespective of the IP3 concentration and IP3R subtype (Foskett et al., 2007). Therefore, if Ca^2+^ entry from stores into the cytosol is limited by IP3R open probability, an increase of the resting [Ca^2+^] directly facilitates Ca^2+^ entry from stores and increases the amplitude of IP3R-driven Ca^2+^ transients. However, the opposite was observed.

Alternatively, a decrease of Ca^2+^ influx from Ca^2+^ stores could also occur at high resting [Ca^2+^] if the latter sufficiently reduced driving forces for Ca^2+^ into the cytosol. Straightforward calculations suggest, however, that, for a Ca^2+^-store membrane potential near zero (Lam and Galione, 2013), the store [Ca^2+^] would have to be ∼200–400 nM (Figure S3D), which is orders of magnitude below experimental estimates of 100–800 μM (Burdakov et al., 2005, Lam and Galione, 2013). For another estimate of the store membrane potential of ∼−85 mV (store negative) (Burdakov et al., 2005), a store [Ca^2+^] of 200–400 μM would reproduce experimental observations (Figure S3F). However, this remains speculative in the absence of direct measurements of the Ca^2+^ store membrane potentials and given the large disparity of theoretical predictions. In addition, equilibration of Ca^2+^ between Ca^2+^ stores and the cytosol at the peak of Ca^2+^ transients could account for lower amplitudes at high resting Ca^2+^ (Figure S4). Interestingly, it was indeed observed recently that rapid and strong reductions of Ca^2+^ levels in the astrocyte endoplasmatic reticulum can occur spontaneously and can also be triggered by norepinephrine (Okubo et al., 2019). Altogether, this indicates that the decrease of Ca^2+^ transient amplitudes with increasing resting Ca^2+^ is mechanistically firmly tied to store-dependent Ca^2+^ signaling. This does not, however, exclude a contribution by other processes. Ca^2+^ removal mechanisms could play a role too. An increased recruitment of Ca^2+^ extrusion at higher Ca^2+^ levels could curtail transients more strongly so that signal amplitudes decrease at higher resting [Ca^2+^].

Throughout most of our study, the peak [Ca^2+^] of transients increased with the resting [Ca^2+^], whereas the amplitude decreased. The previously observed developmental decrease of the astroglial resting [Ca^2+^] (Zheng et al., 2015) could therefore have the opposite effect on astroglial Ca^2+^ signals. However, the signaling pathways driving astroglial Ca^2+^ signals also change during postnatal development (Otsu et al., 2015, Sun et al., 2013), which could mask the effect of age-dependent resting Ca^2+^ on Ca^2+^ transients. Beyond developmental changes, physiological and pathophysiological increases and decreases of the resting [Ca^2+^] and the heterogeneity of resting [Ca^2+^] within and between astrocytes (Agarwal et al., 2017, Jackson and Robinson, 2015, Jennings et al., 2017, Kuchibhotla et al., 2009, Mehina et al., 2017, Shigetomi et al., 2011, Zheng et al., 2015) could be directly translated into corresponding increases and decreases of the peak [Ca^2+^] and changes of the [Ca^2+^] transient amplitude. As a consequence, any cellular process that is driven by astroglial Ca^2+^ increases could be directly controlled by the local resting Ca^2+^ (for review of Ca^2+^-dependent mechanisms, see Araque et al., 2014, Bazargani and Attwell, 2016, Khakh, 2019, Rusakov et al., 2014, and Verkhratsky and Nedergaard 2018). It is an intriguing possibility that astrocytes use their basal Ca^2+^ levels as a “memory trace” of local physiological events. The relationship between basal Ca^2+^ and transient Ca^2+^ signals that we have found could enable astrocytes to convert these cell “memories” into changes of Ca^2+^-dependent cellular activity.

The specific functional consequences will depend on the downstream Ca^2+^-dependent mechanism. For instance, vascular tone and blood flow are controlled by astroglial Ca^2+^ and depend on the magnitude of astroglial Ca^2+^ transients (Lind et al., 2013, Mehina et al., 2017, Mulligan and MacVicar, 2004, Petzold and Murthy, 2011). It is, however, currently not possible to predict how changes of astroglial resting [Ca^2+^] and the resulting shifts of the signal peak [Ca^2+^] affect blood flow and blood vessel diameter, because the quantitative relationship between astroglial [Ca^2+^] and, for instance, blood vessel diameter remains to be firmly determined.

Transmitter release from astrocytes is another interesting Ca^2+^-dependent mechanism. Neuronal neurotransmitter release follows a power law dependence on [Ca^2+^] at many synapses, for example, with an exponent of ∼4 at the Calyx of Held (Schneggenburger and Neher, 2000). A moderate change of the peak [Ca^2+^] therefore results in a relatively larger change of release. In astroglia, the quantitative relationship between [Ca^2+^] and exocytosis has been less intensely studied. In microisland cultures, Ca^2+^ uncaging in astroglia evoked a neuronal current response via glutamate receptors that displayed a dependency on [Ca^2+^] with a Hill coefficient of ∼2 to 3 (Parpura and Haydon, 2000). In addition, the relationship between the rate of membrane capacitance increase after Ca^2+^ uncaging and [Ca^2+^] was approximated by a Hill-type equation with a Hill coefficient of ∼5 in cultured astrocytes (Kreft et al., 2004). Both findings indicate that a moderate increase of astroglial resting [Ca^2+^] and thus the peak [Ca^2+^] of a Ca^2+^ transient could lead to a considerable boost of astroglial transmitter release. However, the relationship between astroglial [Ca^2+^] and astroglial release of a specific transmitter in organized tissue, such as acute slices, or *in vivo* remains to be quantified.

## STAR★Methods

### Key Resources Table

**Table:** 

REAGENT or RESOURCE	SOURCE	IDENTIFIER
**Chemicals, Peptides, and Recombinant Proteins**		

TTX	Tocris	Cat. #1069
DHPG	Tocris	Cat. #0342/1
Phenylephrine	Sigma-Aldrich	Cat. P6126
Ryanodine	Abcam	Cat. AB120083
2-APB	Abcam	Cat. AB120124
Alexa Fluor 594 Hydrazide	ThermoFisher	A10438
Alexa Fluor 488 Hydrazide	ThermoFisher	A10436
Oregon Green 488 BAPTA-1 AM (OGB1-AM)	ThermoFisher	Cat. O6807
Oregon Green 488 BAPTA-2 (OGB2)	ThermoFisher	Cat. O6808
Diazo-2	ThermoFisher	Cat. D3034
NP-EGTA	ThermoFisher	Cat. N6802
Sulforhodamine 101 (SR101)	Sigma-Aldrich	Cat. S7635

**Experimental Models: Organisms/Strains**		

GLASTcreERT2	Mori et al., 2006	N/A
GLASTcreER	Wang et al., 2012	N/A
GCaMP5g-IRES-tdTomato	Gee et al., 2014	N/A
Sprague Dawley	Charles River	#400

**Software and Algorithms**		

Custom FLIM analysis scripts	This manuscript	N/A

### Lead Contact and Materials Availability

Further information and requests for resources and reagents should be directed to and will be fulfilled by the Lead Contact, Christian Henneberger (christian.henneberger@uni-bonn.de). This study did not generate new unique reagents.

### Experimental Model and Subject Details

The experiments outlined below were performed using male Sprague Dawley rats and the transgenic mice lines GLASTcreERT2 (Mori et al., 2006), GLASTcreER (Wang et al., 2012) and GCaMP5g-IRES-tdTomato (Gee et al., 2014). For experiments using transgenic mice, animals of both genders were used to minimize breeding and were randomly assigned to experiments. All experiments and surgical procedures were performed under the required licenses and in full compliance with national, local and European Union regulations. Cre expression was induced by tamoxifen injection (100 mg / kg BW; 1/day IP, 5 days) at the age of 3 weeks unless stated otherwise. The specific age of animals varied between individual experiments and is stated along with other method details below.

### Method Details

#### Hippocampal slice preparation

Acute hippocampal slices from rats and mice were prepared as previously described (Minge et al., 2017). Briefly, acute hippocampal slices were prepared from three to five-week-old male Sprague Dawley rats and six to eight-week-old GLASTcreERT2 x GCaMP5g mice. Slices with a thickness of 350 μm for rats and 300 μm for mice were prepared in an ice-cold slicing solution containing (in mM): NaCl 60, sucrose 105, KCl 2.5, MgCl_2_ 7, NaH_2_PO_4_ 1.25, ascorbic acid 1.3, sodium pyruvate 3, NaHCO_3_ 26, CaCl_2_ 0.5, and glucose 10 (osmolarity 300-305 mOsm), and kept in the slicing solution at 34°C for 15 minutes before being stored at room temperature (21-23°C) in an extracellular solution containing (in mM) NaCl 126, KCl 2.5, MgSO_4_ 1.3, NaH_2_PO_4_ 1.25, NaHCO_3_ 26, CaCl_2_ 2, and glucose 10. All solutions were continuously bubbled with 95% O_2_/ 5% CO_2_. Experiments were performed at ∼34°C.

#### Fluorescence lifetime imaging (FLIM) in acute slices

For astroglial [Ca^2+^] measurement, Oregon Green 488 BAPTA-2 (OGB2, Thermo Fisher Scientifc) was loaded into individual astrocytes as previously described (Henneberger and Rusakov, 2012, Henneberger et al., 2010, Zheng et al., 2015). Briefly, acute slices were transferred into a recording chamber on an upright microscope and held under continuous superfusion with extracellular solution (34°C, continuously bubbled with 95% O_2_/ 5% CO_2_). In these experiments, neuronal activity was inhibited by adding the sodium channel blocker TTX (1 μM, Tocris Bioscience) to the extracellular solution. Putative astrocytes were identified using DIC optics and patched (Multiclamp 700B) with an intracellular solution containing (in mM) KCH_3_O_3_S 135, HEPES 10, di-Tris-Phosphocreatine 10, MgCl_2_ 4, Na_2_-ATP 4, Na-GTP 0.4 (pH adjusted to 7.2 using KOH, osmolarity 290-295 mOsm), OGB2 0.2, and Alexa Fluor 594 0.1 (for visualizing the astrocyte). Cells were held in current clamp or voltage clamp at their resting potential. Astroglial cells were identified by their low resting membrane potential (< −80 mV), low input resistance (< 10 MΩ), symmetric, ‘passive’ responses to current injections (−200, −100, +100, +200 pA) and their characteristic morphology. Recordings were rejected if the access resistance was initially above 20 MΩ or changed more than 30% during the recording. After dye equilibration for 10-20 minutes, Ca^2+^ imaging experiments started. After acquisition of a baseline period, the mGluR agonist DHPG was pressure-applied through a patch pipette placed immediately adjacent to the astrocyte territory. DHPG (300 μM) was dissolved in extracellular solution also containing Alexa Fluor 647 (3 μM) to visualize the ejection of DHPG into the tissue.

Two-photon excitation FLIM was performed as previously described (Minge et al., 2017, Zheng et al., 2015) using time-correlated single photon counting (TCSPC) on Olympus microscopes (FV10MP and FV1000MP) upgraded with TCSPC modules (Picoquant). TCSPC data were collected by frame scanning (∼200x200 pixel corresponding to ∼60x60 μm^2^, 2-3 Hz). The OGB2 fluorescence decay was then analyzed using custom written scripts (MATLAB, Mathworks). The OGB2 fluorescence lifetime was characterized by calculating the ratio of the number of detected photons in two time windows (Figure 1A) and by reconvolution fitting. For the latter, a triple-exponential decay function convolved with the instrument response function (IRF, approximation of experimentally determined IRF) was iteratively fitted to the fluorescence decays (example in Figure S1) and the amplitude-weighted average of the individual decay time constants was calculated: τ = (A1 x τ1 + A2 x τ2 + A3 x τ3) / (A1 + A2 + A3).

Calibration of OGB2 lifetimes were performed in a cuvette as previously described (Minge et al., 2017, Zheng et al., 2015) using a calibration solution based on the intracellular whole-cell patch clamp solution, to which 10 mM BAPTA and 0-10 mM CaCl_2_ and 0.05 μM OGB2 were added. The free [Ca^2+^] was estimated using WebMax Chelator (https://somapp.ucdmc.ucdavis.edu/pharmacology/bers/maxchelator/webmaxc/webmaxcS.htm) taking into account the concentration of Ca^2+^, Ca^2+^-buffers, ATP and Mg^2+^. The relationship between [Ca^2+^] and the photon count ratio and amplitude-weighted average decay constant could be approximated by a Hill function (Figures 1B and S1A). Comparing the two measurements of OGB2 fluorescence decay we found that the results obtained using the photon count ratio varied less especially when the number of analyzed photons was low (Figure S1B). For this reason, the photon count ratio was used for data analysis of physiological experiments and converted into [Ca^2+^]. The OGB2 decay time constants (τ1-3) differed slightly between intracellular measurements and calibrations (Figure S1C). This slight difference was compensated by the following procedure (Figures S1D–S1F). We first estimated the change of the photon count ratio introduced by changes of the decay time constants. This was done by simulating OGB2 fluorescence decays across a set of combinations of component amplitudes (A1-3). For each combination of component amplitudes, the OGB2 fluorescence decay was simulated for intracellular and calibration decay time constants and the photon count ratio was determined for both (Figures S1D and S1E). This relationship was then used to translate photon count ratios obtained from intracellular measurements into calibration photon count ratios and then into Ca^2+^ concentrations (for experimental confirmation see Figure S1F).

For experimental frame scanning TCSPC data, detected photons were pooled across automatically set regions of interest (ROIs, ∼5 × 5 μm^2^) and in time windows of 600 ms (Figures 1D and 2). ROIs were automatically removed from further analysis if the average number of photons per time windows was less than 1500. This effectively removed ROIs outside the investigated cell from analysis. ROIs were removed manually in a second step if they overlapped with either the patch or puff pipette. [Ca^2+^] was then calculated for each time ROI and time window. In experiments with DHPG pressure application, the baseline [Ca^2+^], the response peak [Ca^2+^] and the difference (amplitude) was automatically calculated.

#### Astroglial Ca^2+^ and Ca^2+^ buffer uncaging

In a subset of experiments, FLIM [Ca^2+^] measurements were combined with intracellular Ca^2+^/Ca^2+^ buffer uncaging. In these experiments, either the UV-photolysable Ca^2+^-cage NP-EGTA or the UV-photoactivatable Ca^2+^ buffer diazo-2 was used (5 mM NP-EGTA tetrapotassium salt and 3 mM CaCl_2_ added to the intracellular solution, or 2.5 mM diazo-2 tetrapotassium salt). UV uncaging was performed using a computer-controlled UV-LED (365 nm, Thorlabs) with the output power set to 2.5 mW and activated with 10 pulses of 50 ms delivered at 10 Hz. The UV-stimulus was delivered via a light guide (1 mm, NA 0.39) submerged in the extracellular solution and positioned just above the hippocampal slice in the vicinity of the investigated astrocytes.

#### *In situ* imaging using GCaMP5g

For experiments using the genetically encoded Ca^2+^ indicator GCaMP5g, flox-stop GCaMP5g-IRES-tdTomato mice (Gee et al., 2014) were cross-bred with GLASTcreERT2 mice (Mori et al., 2006) and injected with tamoxifen as described above to induce recombination in astrocytes. Acute slices were prepared from these animals, as explained above, two weeks after the end of tamoxifen injections around postnatal day 60. For recordings, slices were transferred to the recording chamber of an upright microscope as in other *in situ* experiments and the fluorescence intensity of GCaMP5g (G) and tdTomato (T) was recorded by time-lapse frame-scanning (128 × 128 pixels, ∼80 × 80 μm^2^, ∼3 Hz). Image sequences were analyzed in ImageJ (Schneider et al., 2012) by first subtracting the background fluorescence. Spontaneous transients/events were then visually identified and analyzed by placing a ROI (∼3 × 3 μm^2^) centered on the peak elevation of GCaMP5g fluorescence intensity. The ROI fluorescence intensities for each indicator and frame were determined and the fluorescence intensity ration G/T was calculated for each frame. Analogous to FLIM Ca^2+^ imaging, the pre-event G/T, the event peak G/T and the difference between these, i.e., the amplitude were computed as averages over three frames. Because the focus was on the relationship between basal Ca^2+^ levels and the amplitude/peak of Ca^2+^ transients, we did not quantify frequency, spatial extent and propagation of Ca^2+^ transients. For experiments using pressure-applied drugs, a patch pipette containing phenylephrine (PE, 250 μM, Sigma-Aldrich) or ATP (5 mM, Sigma-Aldrich), and also Alexa Fluor 647 (3 μM) for visualization of the pipette and application, was placed in close vicinity of an astrocyte soma (30-40 μm). Only Ca^2+^ transients time-locked to a brief puff (80 ms for PE; 3 × 80 ms at 4 Hz for ATP) were further analyzed.

#### *In vivo* fluorescence lifetime imaging

Two-photon excitation FLIM was performed *in vivo* as previously described (Zheng et al., 2015) and in accordance with the European Commission Directive (86/609/EEC) and the United Kingdom Home Office (Scientific Procedures) Act (1986). Briefly, young male rats (100–120 g) were anesthetized with urethane (initial dose 1.3 g/kg, i.p.; then 10–25 mg/kg/hr, i.v.) following isoflurane (5% in air) induction. The skin overlying the skull was removed and a small craniotomy was made above the somatosensory cortex. Cortical astrocytes were labeled with sulforhodamine 101 (SR101) and OGB1. The solution containing OGB-1 AM (1 mM) and SR101 (to aid identification of astroglia, 25 m M) in artificial cerebrospinal fluid (124 mM NaCl, 3 mM KCl, 2 mM CaCl 2, 26 mM, NaHCO 3, 1.25 mM NaH 2 PO 4, 1 mM MgSO 4, 10 mM D-glucose saturated with 95% O 2 / 5% CO 2 [pH 7.4]) was delivered (volume 1.5 ml) via a glass micropipette to the targeted area of the right primary somatosensory cortex, immediately caudal of the coronal suture. The exposed surface of the cortex was then covered with 1% agarose and protected with a glass coverslip secured to the skull using acrylic dental cement. Two-photon excitation for FLIM acquisition was carried out as described below using a Newport-Spectraphysics Ti:Sapphire Mai-Tai laser, Olympus FV1000 with XLPlan N 25 water immersion multi-photon objective (NA 1.05), and PicoQuant Picoharp 300 TCSPC (alternatively, using Femtonics Femto-2D-FLIM microscope). Frame dimensions were typically 50-70 μm squares which encompasses 3-10 astrocytes in the same field of view. For analysis, see above and Zheng et al. (2015). Continuous frame scans at 0.5 Hz was used for a total duration of 300 s.

#### *In vivo* imaging using GCaMP5g in anesthetized mice

For acute experiments in anesthetized mice, an intensity-based analysis of astroglial Ca^2+^ signaling using the genetically-encoded Ca^2+^ indicator GCaMP5g was performed. Please see above for the induction of Cre recombination in transgenic mice conditionally expressing GLASTcreER (Wang et al., 2012) and GCaMP5g (Gee et al., 2014). In 11 to 14 week old mice, cranial windows were prepared as previously described (Delekate et al., 2014). Briefly, mice were anesthetized with isoflurane (induction, 3% v/v; maintenance, 1%–1.5% v/v) and kept on a heating plate (37°C). The mouse head was fixed in a stereotactic frame, the scalp was removed, and a circular cranial window was drilled above the primary somatosensory cortex. Agarose (1.5%) was placed between the cortex and a cover glass, which was fixed and closed the window. Intensity-based recordings and analyses of astroglial Ca^2+^ signaling were performed as described for *in situ* GCaMP5g experiments.

#### *In vivo* imaging using GCaMP5g during locomotion

For awake *in vivo* imaging, we used mice expressing GCaMP5g and tdTomato in astrocytes as described above. Two weeks after tamoxifen injection (performed in the 10^th^ postnatal week), the chronic cranial window surgery was performed. Briefly, analgesics were administered 30 minutes before surgery start, mice were anesthetized with isoflurane (induction, 3% v/v; maintenance 1%–1.5% v/v) and kept on a heating plate. After fixing the animal’s head in a stereotaxic frame, the scalp was removed, and the skull was pre-treated with light-curable dental cement. A 4 mm circular window was drilled above the primary somatosensory cortex and pre-sealed with a cover glass using super glue. For proper fixation of the window the light-curable dental cement was applied at the edges between the cover glass and the bone. To allow for the head fixation of the animal during the awake imaging, an aluminum head holder was placed at the contralateral side of the skull and fixed with the light-curable cement. After recovery animals were placed back into their home cage and post-surgical care was administered for three consecutive days.

One week after surgery the animals were habituated to run head-fixed on the linear treadmill (Luigs & Neumann, Germany). On the first day animals could explore the treadmill by running freely on the belt. The animals were then kept at one position on the belt by holding their tail and letting them run on the belt for approximately 30 minutes. One day after this initial exploratory phase, the animals were fixed with the head holder into the holding device on the treadmill and allowed to run freely for 30 minutes. This training was repeated for two more days until the animals were running calmly on the belt. On the last day of habituation, the treadmill was positioned under the objective of the multiphoton microscope (Trim ScopeII, LaVision Bio Tec) and the animal was again head-fixed and habituated to the surroundings. The first day of awake *in vivo* imaging took place two weeks after surgery (minimum age of animals was 13 weeks). The animals were head-fixed to the treadmill and allowed to run for a maximum of 60 minutes while being imaged. Consecutive imaging series were taken for 10 minutes each with a 16x objective (Nikon). Image acquisition was performed at ∼3 Hz, with a pixel dwell time of ∼2 μs, a frame size of ∼200 μm x 200 μm and a nominal resolution of 0.5 μm/pixel. To correct for movement artifacts, we made use of a custom-written MATLAB (Mathworks) script to register and align imaging data in an elastic group-wise fashion. The script uses a published algorithm (Guizar-Sicairos et al., 2008) with an additional implementation of the Lucas-Kanade algorithm. The field of view contained two to three astrocytes, whose Ca^2+^ transients were pooled for the analysis. The movement of the animal was detected by a digital position readout device (Luigs&Neumann, Germany). The velocity of the animal moving on the belt was acquired at 20 Hz starting simultaneously with the start of image acquisition. Output data was then further processed to correlate movement and imaging data. On average the animals moved with a speed of 65.4 ± 18.3 cm/min. To quantify locomotion-associated Ca^2+^ transients, the change in position was calculated for time windows of three seconds. All transients whose peak occurred within ± 6 s of a period of locomotion with a speed higher than 1 cm/s were included in the analysis.

### Quantification and Statistical Analysis

Numerical data are reported as mean ± s.e.m. with n being the number of samples. In figures, asterisks indicate statistical significance (details in figure, figure legend or text). In Box and Whisker plots the box indicates the 25^th^ and 75^th^ percentile, whiskers 5% and 95% percentiles, the horizontal line in the box the median and the filled circle the mean. Individual data points are displayed as hollow circles on top of the Box and Whisker plot. Student’s t tests, Spearman’s rank correlation (Spearman rank correlation coefficient, R) and other statistical tools were used as indicated and performed in MATLAB (Mathworks) and Origin (OriginLab). Only two-sided tests were used. p represents the level of significance. Significance levels are indicated in figures by asterisks (^∗^ p < 0.05, ^∗∗^ p < 0.01, ^∗∗∗^ p < 0.001) unless stated otherwise. ‘n’ indicates the number of independent samples and refers to, unless indicated otherwise, to the number of individual astrocyte recordings. For acute slices, on average one recording was performed per slice and one to two successful recordings were obtained per animal.

To establish the dependence of astrocytic [Ca^2+^] transient peaks and amplitudes on the local basal [Ca^2+^] within individual cells, a measure of correlation is needed. Since the exact mathematical relationship between these parameters is not known *a priori* and may also differ between cells, a non-parametric approach was required. We used Spearman’s rank correlation coefficient (R) to extract a single and unbiased measure that can be used across different cells, techniques and preparations. In this analysis, Spearman’s R is the biological relevant statistical unit (e.g., in an individual cell). It is a dimensionless variable reporting the degree of x-y correlation. In our case, it captures how well the relationship between the basal [Ca^2+^] and the peak/amplitude of Ca^2+^ transients can be described using a monotonic function (+1 = always increases with resting [Ca^2+^], −1 = always decreases with resting [Ca^2+^]). See Figures 1F and 3B for examples. This measure isolates the overall dependence of peak and amplitude on resting [Ca^2+^] and enabled us to compare different cells irrespective of the precise values, which can vary considerably from one cell to another. The overall average dependence of peak and amplitude on resting [Ca^2+^] across a set of cells was then calculated as the mean ± s.e.m. and displayed together with individual data points in boxplots (see above and Figures 1G, 2E, 3C, 3E–3H, 4B, 4C, 5C, and 6E). Two-tailed, one population Student’s t tests versus zero were used to establish if Spearman rank correlation coefficients across the entire population of recordings were significantly different from zero.

Estimates for the calculations presented in Figure S4 were obtained from the literature (De Young and Keizer, 1992, Höfer et al., 2002, Patrushev et al., 2013). In image panels not showing a color scale for Ca^2+^ concentrations, fluorescence intensities are color-coded using ImageJ’s color lookup tables (*red hot* and *green fire blue*, see Figure 5 for an example).

### Data and Code Availability

The datasets and custom code supporting the current study have not been deposited in a public repository but are available upon reasonable request from the corresponding author.

## Supplemental Information

Document S1. Figures S1–S6

Document S2. Article plus Supplemental Information
