# Supplementary material for: Local Resting Ca2+ Controls the Scale of Astroglial Ca2+ Signals
Source: Cell Rep. 2020 Mar 10;30(10):3466–3477.e4. doi: 10.1016/j.celrep.2020.02.043 (PMC7068654; doi:10.1016/j.celrep.2020.02.043)
Supplement: Document S1. Figures S1–S6 [file mmc1.pdf]

Cell Reports, Volume *30*

Supplemental Information

Local Resting Ca^{2+} Controls

the Scale of Astroglial Ca^{2+} Signals

Claire M. King, Kirsten Bohmbach, Daniel Minge, Andrea Delekate, Kaiyu Zheng, James Reynolds, Cordula Rakers, Andre Zeug, Gabor C. Petzold, Dmitri A. Rusakov, and Christian Henneberger

Figure S1

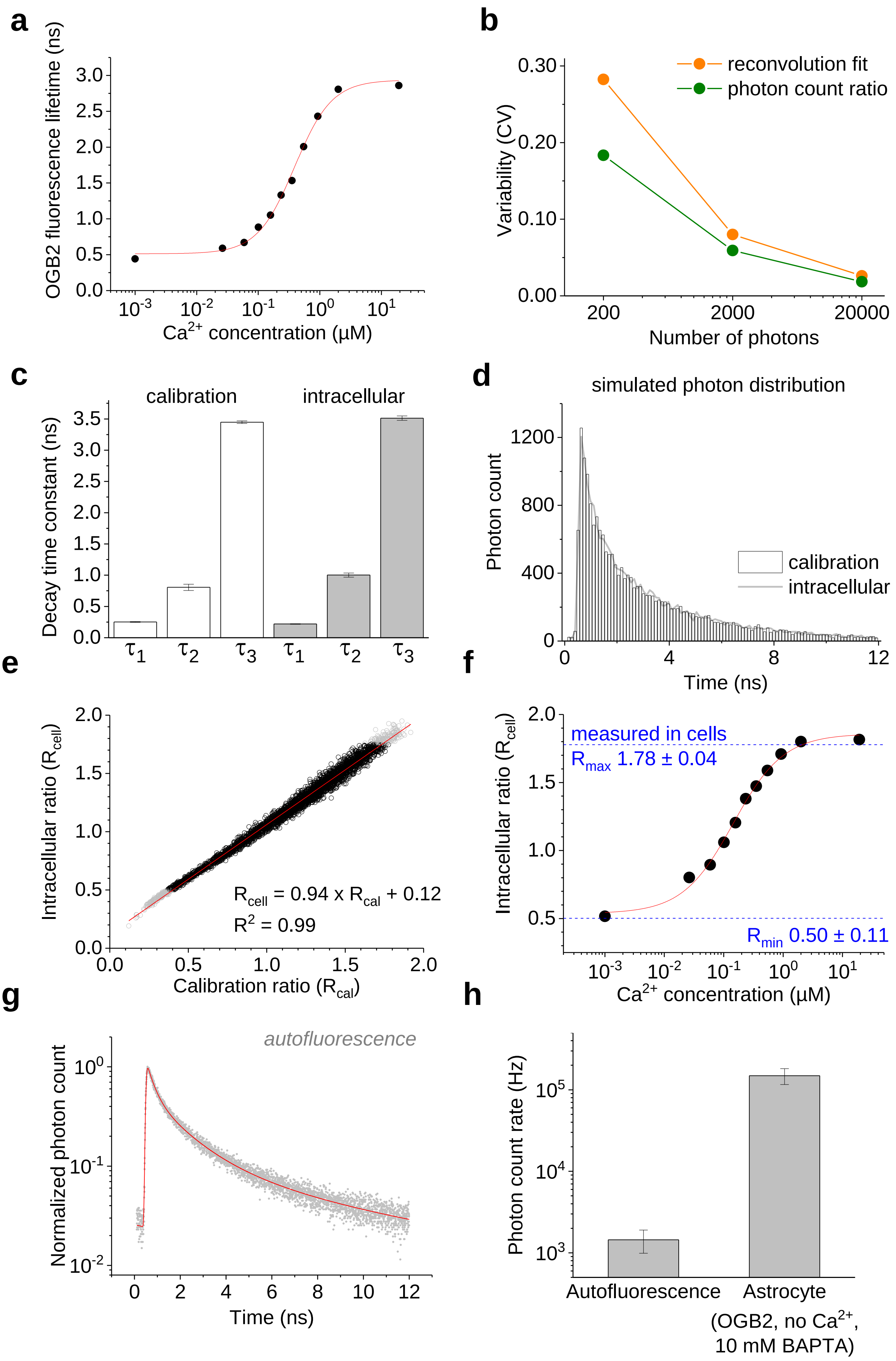
a
OGB2 fluorescence lifetime (ns)
Ca2+ concentration (µM)
b
reconvolution fit
photon count ratio
Variability (CV)
Number of photons
c
calibration
intracellular
Decay time constant (ns)
τ1
τ2
τ3
d
simulated photon distribution
Photon count
calibration
intracellular
Time (ns)
e
Intracellular ratio (Rcell)
Rcell = 0.94 x Rcal + 0.12
R2 = 0.99
Calibration ratio (Rcal)
f
measured in cells
Rmax 1.78 ± 0.04
Rmin 0.50 ± 0.11
Intracellular ratio (Rcell)
Ca2+ concentration (µM)
g
autofluorescence
Normalized photon count
Time (ns)
h
Photon count rate (Hz)
Autofluorescence
Astrocyte
(OGB2, no Ca2+,
10 mM BAPTA)

FLIM data analysis: comparison of reconvolution fitting and photon count ratios, comparison of OGB2 fluorescence decay between cuvette calibration and intracellular recordings, negligible contribution of auto-fluorescence to FLIM Ca^{2+} measurements (related to Figure 1).

a) In addition to analysing data using photon count ratios (Fig. 1a-b), reconvolution fitting of FLIM calibration data was performed for comparison. Lifetime data (Fig. 1a, time-resolved OGB2 fluorescence) was approximated with a triple exponential decay function convolved with the instrument response function (IRF) of the imaging system. For each [Ca^{2+}], the amplitude-weighted average fluorescence decay time constant was calculated. The relationship between this time constant and [Ca^{2+}] was approximated by a Hill function (R^2 = 0.99).

b) The sensitivity of both methods to the number of measured photon detection times was quantified. For this test, lifetime data sets were generated by random sampling of 200, 2000 or 20000 photon detection times from a reference lifetime calibration measurement and each data set was analysed using both methods (1000 data sets for each number of photon detection times). The coefficients of variation (CV) of the amplitude-weighted decay time constant and the photon count ratios were calculated across the data sets for 200, 2000 and 20000 sampled photon detection times. Intuitively, the variability of analysis results decreased the more photon detection times were sampled to build a lifetime curve. Especially for fewer photon detection times, the CV of photon count ratios was smaller than the CV of time constants. Thus, analysis of photon count ratios provides more stable results when few photon detection times are available for analysis.

c) The time-resolved OGB2 fluorescence decay was iteratively fitted by reconvolution of a triple-exponential decay function with the IRF (see also Material and Methods). Slightly different decay time constants ($\tau_{1...3}$) were obtained from calibration data and intracellular recordings (n = 7 for cuvette calibrations and 13 intracellular recordings from astrocytes, respectively).

d) The significance of the lifetime component differences was estimated. Experimental fluorescence decay profiles were simulated by randomly choosing the three amplitudes of individual components and convolving the resulting triple-exponential decay function with the IRF. The convolved result was then transformed into a probability density function, which gives the probability distribution of photon detection times for the chosen set of component amplitudes. A simulated fluorescence profile was then obtained by randomly sampling 20000 photons from this distribution. For a given set of component amplitudes, this was performed with the lifetime decay time constants for cuvette calibration (bars) and intracellular recordings (line).

e) A total number of 10000 photon distribution pairs were simulated (black circles for experimentally observed range and grey circles outside). A strong linear relationship between the simulated intracellular and cuvette calibration photon count ratios was observed. This linear relationship was used to transform the ratio obtained from intracellular recordings to an equivalent ratio from the cuvette calibration and, thus, for conversion of experimentally observed photon count ratios into Ca^{2+} concentrations.

f) The relationship between [Ca^{2+}] and intracellularly measured photon count ratio is displayed. The minimum (R_{min}) and maximum (R_{max}) were verified experimentally. For R_{min}, 10 mM BAPTA were added to the intracellular solution (n = 3 astrocytes). For R_{max}, 10 mM BAPTA and 10 mM $CaCl_2$ were added to the intracellular solution (saturating free Ca^{2+} of 19.2 µM, n = 4). Note the match between the experimentally determined range and the calibration.

g) A number of molecules present in brain tissue can fluoresce in response to two-photon excitation. We tested if such autofluorescence contributes significantly to our measurements by imaging tissue at comparable depth, laser power and imaging settings (n = 3). An example of the observed time-resolved autofluorescence is shown (grey dots) together by its approximation by reconvolution fitting (triple exponential decay). The amplitude-weighted average lifetime was 0.96 ± 0.076 ns (n =3).

h) To estimate the contribution of autofluorescence to [Ca^{2+}] measurements, we compared the rate at which autofluorescence photons were detected with the rate from otherwise identical experiments in which astrocytes were filled with OGB2, the Ca^{2+} buffer BAPTA (10 mM) without any added Ca^{2+}. Under these conditions of minimal OGB2 fluorescence, the detection rate of autofluorescence photons was two orders of magnitude lower than in astrocyte recordings indicating that the contribution of autofluorescence is negligible. To quantify the error introduced into [Ca^{2+}] measurements by autofluorescence we generated synthetic lifetime curves (random photon detection time sampling, see above) of OGB2 for [Ca^{2+}] = 101 nM and added similarly generated autofluorescence with 100x fewer photon detection times. The error was then estimated by analysing [Ca^{2+}] using photon count ratios for generated OGB2 and OGB2+autofluorescence lifetime profiles. It was -1.1 ± 0.83 % (n = 100).

Figure S2

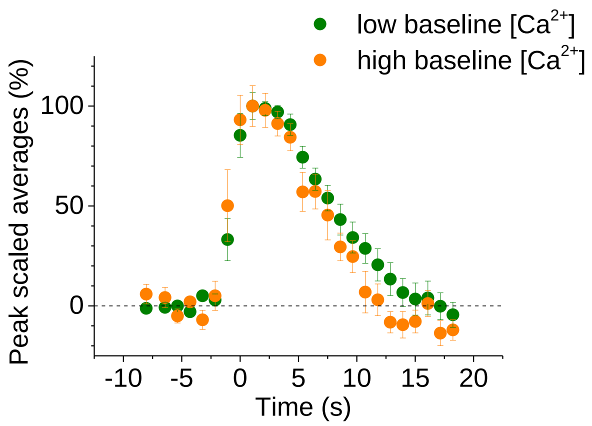

Kinetics of DHPG-induced Ca^{2+} transients are not controlled by resting [Ca^{2+}] (related to Figure 1).

Ca^{2+} transient kinetics were analysed for the data from Fig. 1. For each cell, [Ca^{2+}] transients from ROIs with baseline [Ca^{2+}] below mean (low baseline [Ca^{2+}], green) and above (high baseline [Ca^{2+}], orange) were averaged and then peak-scaled. These were averaged across all recordings (n = 11).

Figure S3

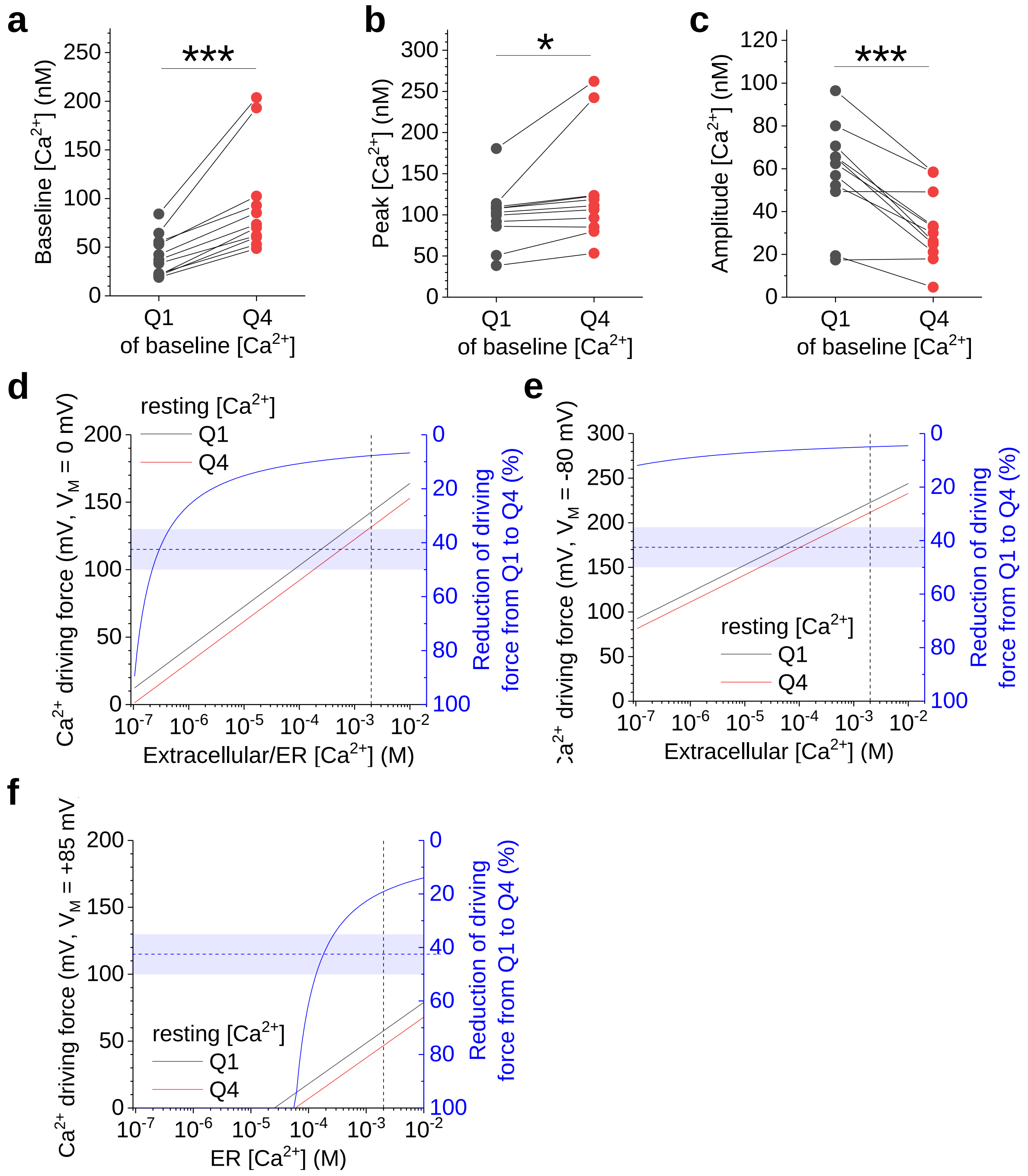

Comparison of [Ca^{2+}] transient properties and Ca^{2+} driving forces between low- and high-baseline regions of interest (related to Figure 1).

To confirm findings obtained using Spearman rank coefficients (Rs), we directly compared peak, amplitude and baseline of DHPG-induced [Ca^{2+}] transients (same data as in Fig. 1) in regions of interest (ROIs) with low and high baseline [Ca^{2+}]. For each cell (n = 11), ROIs with a baseline [Ca^{2+}] in the 1st quartile (Q1) and in the 4th quartile (Q4) were identified. We then calculated for each cell the average baseline, peak and amplitude of [Ca^{2+}] transients in both

quartiles (Q1 and Q4). Please note, that in this analysis 50% of ROIs are not taken into account by definition.

a) As expected, the baseline is significantly higher in Q4 compared to Q1 ($p = 5.3 \times 10^{-4}$, paired Student's t-test).

b) The peak [Ca^{2+}] is significantly higher when the baseline [Ca^{2+}] is high ($p = 0.041$, paired Student's t-test).

c) The amplitude of [Ca^{2+}] is significantly lower when the baseline [Ca^{2+}] is high ($p = 2.8 \times 10^{-4}$, paired Student's t-test).

d) Estimate of the Ca^{2+} driving force for Q1 and Q4 baseline [Ca^{2+}] and their ratio, and comparison to experimentally observed reduction of [Ca^{2+}] transient amplitudes. We calculated the Nernst equilibrium potential for a range of Ca^{2+} concentrations outside of the astrocyte cytosol (extracellular or endoplasmic reticulum, ER) (black left y-axis) for the average baseline [Ca^{2+}] in Q1 (41.3 ± 6.36 nM, black line) and in Q4 (95.0 ± 16.2 nM, red line) and subtracted the membrane potential (V_M, 0 mV here). The reduction of the driving force from Q1 to Q4 is shown in percent (blue line and right blue y-axis). At the nominal extracellular [Ca^{2+}] of 2 mM, the change of the equilibrium potential from Q1 to Q4 is < 10 % (dashed black vertical line). The horizontal blue dashed line and the transparent blue bar indicate the experimentally observed [Ca^{2+}] transient amplitude reduction (by 42.5 ± 7.70 %).

Under these conditions and assuming that Ca^{2+} influx into the astrocyte cytosol is directly proportional to the driving force, the observed reduction of Ca^{2+} transient amplitudes could be explained by a decrease of the driving force if [Ca^{2+}] outside the cytosol (extracellular space or ER) was as low as ~ 200-400 nM (intersection of dashed and solid blue lines).

e) Comparison of driving forces for Ca^{2+} across the astroglial plasma membrane at a transmembrane potential of -80 mV. The difference between driving forces between Q1 and Q4 basal [Ca^{2+}] is < 15 % over the entire range of calculated extracellular [Ca^{2+}]. Thus, the decrease of the Ca^{2+} driving force at high basal cytosolic [Ca^{2+}] cannot explain the reduction of amplitudes if they are generated entirely by Ca^{2+} entry from extracellular space.

f) Precise measurements of the astroglial ER membrane potentials are not available to our knowledge. Estimates of the ER membrane potential V_{ER} are commonly based on theoretical considerations and range from, more recently, close to zero (Lam and Galione, 2013) to previously ~ -74 to -90 mV (ER-negative) (Burdakov et al., 2005). If V_{ER} is close to zero, an ER [Ca^{2+}] of 200-400 nM would be required to explain the amplitude decrease (see **d**). If V_{ER} is close to +85 mV however (cytosol positive, ER negative), a store [Ca^{2+}] of 200-400 µM can explain the reduced amplitudes at high basal [Ca^{2+}], which is in line with estimates of ER [Ca^{2+}] of 100-800 µM (Burdakov et al., 2005; Lam and Galione, 2013).

Figure S4

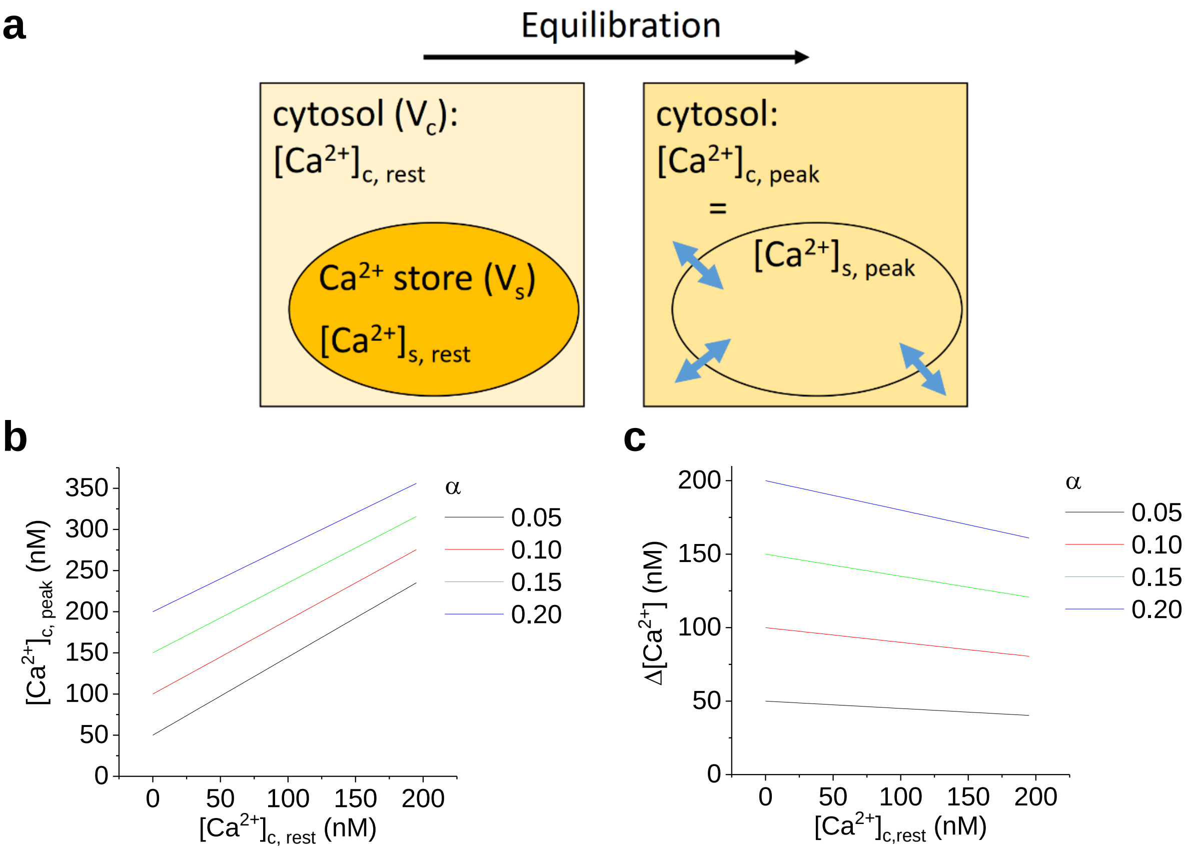

Equilibration of Ca^{2+} between cytosol and Ca^{2+}-stores during astroglial Ca^{2+} transients reproduces the experimentally observed dependence of transient peak $[Ca^{2+}]$ and amplitude on resting $[Ca^{2+}]$ (related to Figures 1-4).

a) Simplified astroglial compartment containing a Ca^{2+} store. The cytosolic $[Ca^{2+}]$ after equilibration between both compartments, at the Ca^{2+} transient peak, is given by

$$[Ca^{2+}]_{c,\ peak} = (V_c \times [Ca^{2+}]_{c,\ rest} + V_s \times [Ca^{2+}]_{s,\ rest}) / (V_c + V_s),$$

where the subscripts c and s denote the cytosolic and Ca^{2+}-store volume and $[Ca^{2+}]$, respectively. Ca^{2+} extrusion mechanisms are ignored for the sake of simplicity. We next define α to be the fraction of the total volume ($V = V_c + V_s$) occupied by Ca^{2+} stores. Substituting V_s by $\alpha \times V$ and V_c by $(1 - \alpha) \times V$ yields

$$[Ca^{2+}]_{c,\ peak} = (1 - \alpha) \times [Ca^{2+}]_{c,\ rest} + \alpha \times [Ca^{2+}]_{s,\ rest}.$$

The $[Ca^{2+}]$ transient amplitude is then given by

$$\Delta[Ca^{2+}]_c = [Ca^{2+}]_{c,\ peak} - [Ca^{2+}]_{c,\ rest} = \alpha \times [Ca^{2+}]_{s,\ rest} - \alpha \times [Ca^{2+}]_{c,\ rest}.$$

b) Relationship between the cytosolic resting $[Ca^{2+}]_{c,\ rest}$ and the Ca^{2+} transient peak $[Ca^{2+}]$ over a range of Ca^{2+} store volume fractions (α, 0.05 - 0.20) (De Young and Keizer, 1992; Höfer et al., 2002; Patrushev et al., 2013) and assuming a store $[Ca^{2+}]$ of 1 μM.

c) Same as **b** for the Ca^{2+} transient amplitude. See Fig. 1 for comparison. Note that the slope is set by the Ca^{2+} store volume fraction. As a consequence, the strength of the negative correlation between resting [Ca^{2+}] and [Ca^{2+}] transient amplitude is expected to vary within a single astrocyte depending on the local abundance of Ca^{2+} stores.

Figure S5

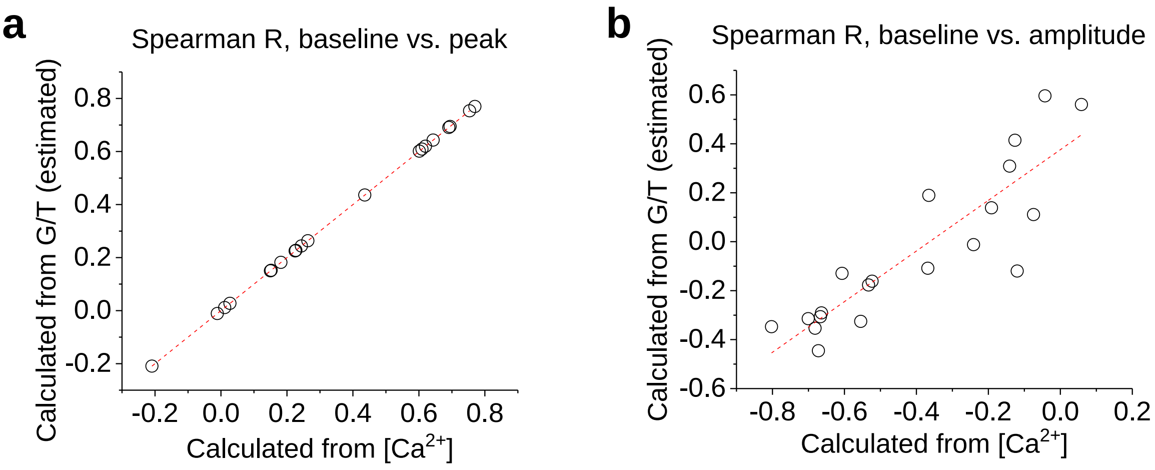

Correction of Spearman correlations obtained from recordings using GCaMP5g (related to Figure 4).

The fluorescence intensity of GCaMP5g is non-linearly correlated with [Ca^{2+}]. Its dependence on [Ca^{2+}] has been approximated with a Hill function with a K_d of 460 nM and a Hill coefficient of 2.5 (Akerboom et al., 2012). How this nonlinearity affects Spearman rank correlations between baseline and amplitude/peak of Ca^{2+} transients was investigated as follows. For each [Ca^{2+}] transient in each FLIM recording of Fig. 1 and 3, the corresponding fraction of Ca^{2+}-bound GCaMP5G was calculated for the baseline and peak [Ca^{2+}] of the transient. These fractions are linearly correlated with the corresponding fluorescence intensity ratio of GCaMP5G/tdTomato (G/T) within a single experiment and cell. The amplitude (ΔG/T) of each event was calculated as the difference between peak G/T and baseline G/T. We then calculated Spearman Rs between baseline (G/T) and peak (G/T) and between baseline (G/T) and amplitude (ΔG/T) and compared these to the Spearman Rs obtained from direct [Ca^{2+}] measurements.

a) For correlations between baseline and peak, we observed a direct linear dependence between the Spearman R's obtained from estimated G/T data and [Ca^{2+}] data (n = 20, R^2 = 1, slope = 1, y-intercept = 0). Therefore, no corrections are needed for Spearman rank correlation coefficients obtained between baseline G/T and peak G/T.

b) For correlations between baseline and amplitude, we again obtained a direct linear dependence between Spearman Rs calculated from estimated G/T data and [Ca^{2+}] (n = 20, R^2 = 0.78). The y-intercept differed significantly from zero (y-intercept = 0.38 ± 0.062, p = 1.1 x 10^{-5}) and the slope was 1.03 ± 0.13. Therefore, we corrected Spearman Rs calculated between baseline and amplitude in GCaMP5g/tdTomato recordings by subtracting 0.38 in order to account for the non-linear dependence of GCaMP5g fluorescence on [Ca^{2+}].

Figure S6

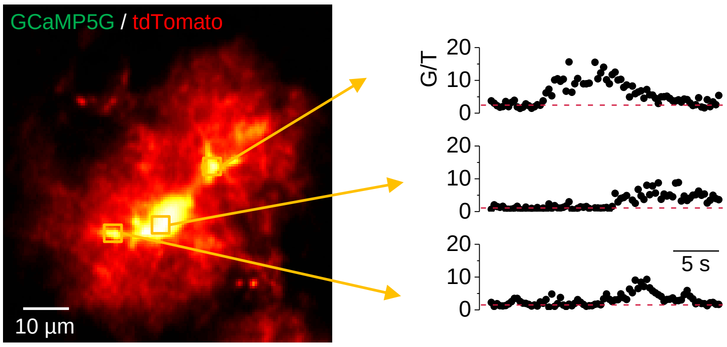

Second example of an *in vivo* recording in awake animals (related to Figure 6).

Please see legend of Fig. 6 for details.
